# A novel hybrid design and modelling of a customised graded Ti-6Al-4V porous hip implant to reduce stress-shielding: An experimental and numerical analysis

**DOI:** 10.3389/fbioe.2023.1092361

**Published:** 2023-01-26

**Authors:** Seyed Ataollah Naghavi, Maryam Tamaddon, Pilar Garcia-Souto, Mehran Moazen, Stephen Taylor, Jia Hua, Chaozong Liu

**Affiliations:** ^1^ Institute of Orthopaedics and Musculoskeletal Science, Division of Surgery and Interventional Science, Royal National Orthopaedic Hospital, University College London, Stanmore, United Kingdom; ^2^ Medical Physics and Biomedical Engineering, University College London, London, United Kingdom; ^3^ Department of Mechanical Engineering, University College London, London, United Kingdom; ^4^ School of Science and Technology, Middlesex University, London, United Kingdom

**Keywords:** additive manufacturing, hip implant, stress shielding, bone resorption, aseptic loosening, hip stiffness, finite element analysis, porous implant

## Abstract

Stress shielding secondary to bone resorption is one of the main causes of aseptic loosening, which limits the lifespan of hip prostheses and exacerbates revision surgery rates. In order to minimise post-hip replacement stress variations, this investigation proposes a low-stiffness, porous Ti6Al4V hip prosthesis, developed through selective laser melting (SLM). The stress shielding effect and potential bone resorption properties of the porous hip implant were investigated through both *in vitro* quasi-physiological experimental assays, together with finite element analysis. A solid hip implant was incorporated in this investigation for contrast, as a control group. The stiffness and fatigue properties of both the solid and the porous hip implants were measured through compression tests. The safety factor of the porous hip stem under both static and dynamic loading patterns was obtained through simulation. The porous hip implant was inserted into Sawbone/PMMA cement and was loaded to 2,300 N (compression). The proposed porous hip implant demonstrated a more natural stress distribution, with reduced stress shielding (by 70%) and loss in bone mass (by 60%), when compared to a fully solid hip implant. Solid and porous hip stems had a stiffness of 2.76 kN/mm and 2.15 kN/mm respectively. Considering all daily activities, the porous hip stem had a factor of safety greater than 2. At the 2,300 N load, maximum von Mises stresses on the hip stem were observed as 112 MPa on the medial neck and 290 MPa on the distal restriction point, whereby such values remained below the endurance limit of 3D printed Ti6Al4V (375 MPa). Overall, through the strut thickness optimisation process for a Ti6Al4V porous hip stem, stress shielding and bone resorption can be reduced, therefore proposing a potential replacement for the generic solid implant.

## 1 Introduction

Total hip arthroplasty (THA) is one of the most common orthopaedic procedures, whereby the damaged hip is either partially or totally replaced with an implant (Pivec et al., 2012). THA is an effective treatment for hip fracture, osteoarthritis, rheumatoid arthritis, osteomyelitis and osteonecrosis of the femoral head (Alonso-Rasgado et al., 2018). Annually, over a million patients are successfully treated worldwide by THA, this number is expected to double in the next two decades (Pivec et al., 2012). This is mainly due to a global increase in the ageing population, which raises the demand for THA procedures (Windler and Klabunde, 2001). When designing load-bearing implants, such as hip implants, long-term survival is one of the main criteria during the design phase (Kayabasi and Ekici, 2008). Despite the high clinical success rate (95% within 10 years) for THA, over 15% of patients still require revision surgery, where 50% of the revisions are undertaken within 5 years of initial surgery with 33% due to instability and 24% resulting from infection (Ulrich et al., 2008). The majority of currently available hip implants last for approximately 25 years for elderly patients and 10–15 years for younger patients, depending on the patient’s activity level and lifestyle, implant type, fixation method, and implant material (Harrysson et al., 2008; Evans et al., 2019). Some of the major concerns and complications concerning currently available commercial hip implants and THA include stress shielding effect, bone resorption, aseptic loosening, thigh pain, and peri-prosthetic fracture that may lead to revision surgery of the hip implant (Pivec et al., 2012). Hip implants are made of various materials, such as titanium-based or cobalt-chromium alloys, 316 L stainless steel and tantalum, where all are considerably stiffer (110–230 GPa) than the surrounding cortical bone (<30 GPa) (Arabnejad Khanoki and Pasini, 2012). Following hip arthroplasty, a considerable level of mechanical loading is naturally transferred to the hip stem, shielding the stress that would have been transferred to the femoral bone (Wang et al., 2018). This is known as the stress shielding effect. Stress shielding is a well-established issue for proximal femoral bone for several decades and is still attracting significant research interest (Liu et al., 2021a; Guo et al., 2022; Huo et al., 2022).

According to Wolff’s law, bone reconstructs and self-organizes its topology to adapt to the external load that is being exerted onto it. This is followed by bone re-modelling which results in either bone formation or resorption (Huiskes et al., 2000). In the case of stress shielding, a large portion of the natural load is removed from the cortical bone, resulting in a loss of the mechanical stimulus that drives bone formation, leading to bone loss over time (Tan and van Arkel, 2021). This consequently weakens the implant support and increases the risk of elevated micromotion at the interface of implant and bone, leading to aseptic loosening of the implant. Implant loosening can cause thigh pain, increase the risk of peri-prosthetic fracture, and eventually, lead to revision surgery (Wang et al., 2020). Micromotions >200 μm are widely suggested to promote the formation of fibrous connective tissues, i.e., inhibiting osseointegration to the implant. This consequently reduces the long-term stability of the implant (Caouette et al., 2011).

In order to prevent the main cause of bone resorption and aseptic loosening, the issue has to be resolved ‘in the bud’, through the development of a hip implant that has a stiffness similar to the actual femoral bone. Hip implant stiffness can either be reduced by selecting materials of different properties, or through geometrical modifications including geometric profile modifications (shortening stem length, adding a collar to the stem or matching geometry to the proximal femoral canal), or a combination of both (Pivec et al., 2012; Liu et al., 2021a). For example, Sabatini and Goswami (Sabatini and Goswami, 2008) enhanced the stress distribution field by using elliptical and circular cross-sections instead of trapezoidal sections, while Gross and Abel (Gross and Abel, 2001) developed a hollow hip stem, both studies reporting a reduced stress shielding in the bone. However, it is known from the literature that geometric modifications alone are not sufficient to achieve a realistic stiffness profile as present within the femoral bone (De Santis et al., 2000). Previously, multiple materials were considered to reduce hip implant stiffness, and this has led to the development of ‘isoelastic stems’. Composite materials based on carbon fibre, polyetheretherketone (PEEK), glass fibre and polyethyleneimine (PEI) have been also explored as potential candidates for hip implants (Brandwood et al., 1992; De Santis et al., 2000; Srinivasan et al., 2000; Bougherara et al., 2011; Naghavi et al., 2022a). Although they have been shown to reduce the stiffness profile of the hip implant, the results have not been promising (Scholz et al., 2011; Saad et al., 2018). For example, carbon fibre implants can lead to macrophage proliferation, which can be transferred into the lymphatic system, resulting in an undesired systemic circulation in the patients (Brandwood et al., 1992; Scholz et al., 2011; Saad et al., 2018). It is known from the literature that composite hip stems also frequently fail due to a lack of bonding strength at the implant-bone interface (Harrysson et al., 2008).

Apart from solid implants, porous implants have also gained interest in the orthopaedic trauma community. Such implants promote bone tissue ingrowth and can enhance long-term implant fixation (Ryan et al., 2006). The manufacturing of micro-scale lattice structures is currently possible due to advances in metal additive manufacturing (AM) technologies, such as selective laser melting (SLM), selective laser sintering (SLS), and electron beam melting (EBM) (Wang et al., 2018). The benefits of AM scaffolds include a high level of accuracy and reliability. By having a high degree of control over the AM process, it is now possible to produce customised hip stems with graded lattice structures, resulting in stems with lower stiffness profiles than their solid counterparts (Arabnejad Khanoki and Pasini, 2012). Furthermore, porous hip stems can enhance bone ingrowth within a stem, resulting in hip stem long-term stability. However, one major drawback of producing a porous hip stem is its reduced strength. Therefore, a compromise between stiffness, strength and porosity is required when designing a porous hip stem. The aim is to produce a hip stem which has a stiffness comparable to the femoral bone, together with elevated strength in order to withstand the fatigue (defined as 5 million cycles at 2,300 N load based on ISO 7206-4:2010) and have a minimum porosity of 50% for enhanced osseointegration (Arabnejad et al., 2016; Sun et al., 2022a). Table 1 shows the amount of stress shielding and bone resorption reduction based on approaches in recent studies on porous hip stems. Generally, it is shown that stress shielding is reduced by around 17%–32%, resulting in a reduced bone resorption by 40%–75%.

**TABLE 1:** Recent studies considering porous hip stem to reduce stress shielding effect and bone loss.

Study by	Approach	Benefit
Arabnejad et al. (2017)	Porous tetrahedron hip stem	Bone loss reduction by 75%. 86% in Gruen zone 6. 40% in Gruen zone 7
Wang et al. (2018)	Porous tetrahedron hip stem	Bone loss reduction by 58%
Sun et al. (2018)	Gradient modulus distribution	Bone loss reduction by 40%. Safety factor of 11.3
Sun et al. (2022b)	Porous graded body-centered-cube hip stem	Minimizes stress shielding Guarantees safety factor
Kladovasilakis et al. (2020)	Topology optimisation of TPMS structures	Safety factor of 2.08
Gao et al. (2022)	Kriging approximation	Stress shielding reduction by 17%
Jette et al. (2018)	Porous diamond hip stem	Stiffness reduction by 31%
Abate et al. (2021)	Porous vintiles hip stem	Stiffness reduction by 62%
Mehboob et al. (2020b)	Porous body-centered-cube hip stem	Stress shielding reduction by 28%. Stiffness reduction by 26%
Tan and van Arkel (2021)	Topology optimisation by upon stochastic porous structure and a selectively hollowed approach	Stress shielding reduction by: 15% in Gruen zone 6. 25% in Gruen zone 7. Stiffness reduction by 40%
He et al. (2018)	Porous Face and Body Centered Cubic with Vertical Struts Unit Cell	Stress shielding reduction by 57%
Yan et al. (2010)	Porous titanium hip stem with different porosities	Bone loss reduction by 80% with 20% porosity. Bone loss reduction by 92% with 60% porosity
Wang et al. (2020)	Porous diamond cubic hip stem.-Distally increased porosity axial gradient.-Inward increased porosity radiant gradient	Bone loss reduction by 74%
Al Zoubi et al. (2022)	Porous cube hip stem	Stress shielding reduction by: 22% in Gruen zone 6. 65% in Gruen zone 7
Cortis et al. (2022)	Porous body-centered-cube hip stem	Stress shielding reduction by: 11% in Gruen zone 6. 25% in Gruen zone 7

Triply periodic minimal surface (TPMS) and lattice structures have gained significant attention in the biomedical field, especially in tissue engineering scaffolds, due to their ability to provide an enhanced cell migration rate, together with having elevated structural stiffness (Kapfer et al., 2011; Zhu et al., 2020; Naghavi et al., 2022b). According to the literature, amongst the currently developed TPMS structures, the Schoen Gyroid structure was deemed to have the best geometry for enhanced bone cell migration and high mechanical strength, and Schwarz Diamond was found to have increased mechanical strength (Al-Ketan and Abu Al-Rub, 2019; Timercan et al., 2021).

The evaluation of the long-term survival of a porous hip stem is based upon several factors, including stress shielding, fatigue strength, and bone ingrowth (Liu et al., 2021a; Liu et al., 2021b). When performing finite element analysis (FEA) of the hip stem, many studies consider applying equivalent material properties (apparent elastic modulus) of porous structures to solid hip stem (Arabnejad et al., 2017; Mehboob, et al., 2020a) in order to reduce computational time, without the need for high-performance computers. The validity of this approach is disputed; while Jette et al. identified consistency between the FEA results and that of *in vitro* mechanical testing (Jetté et al., 2018). Simoneau et al. demonstrated that the strain field of the simplified model did not match the data measured by digital image correlation technology (Simoneau et al., 2017). Another disadvantage of using equivalent material properties in replacement of porous structure is that local stresses on the struts and contacts between the porous stem and femoral bone cannot be reliably evaluated, which can consequently mislead to erring conclusions.

The aim of this study was to develop an optimized, functionally graded hybrid (gyroid-diamond) Ti6Al4V alloy porous hip stem, which has–in concomitance–overall stiffness profile within the range of femoral bone, elevated strength to withstand 5 million cycles, and a porosity profile that is optimal for enhanced osseointegration. Current approaches in the literature do not often consider the amount of stress shielding and bone resorption in Gruen zones while actually they are very important for clinicians when evaluating the performance of a hip implant. Therefore, the approach used in this investigation included the study of both these parameters using experimental testing and numerical simulations for both the artificial intact femur (Sawbone) and the implanted artificial femurs (using a solid and porous hip stem with identical geometries) for compression. This study hypothesizes that comparing a generic hip stem, to the proposed porous hip stem will effectively reduce the stress shielding effect and bone resorption. The performance of the porous hip stem is evaluated through stress shielding and bone resorption reduction in all Gruen zones.

## 2 Materials and methods

### 2.1 Hip implant design and manufacturing

**FIGURE 1:**
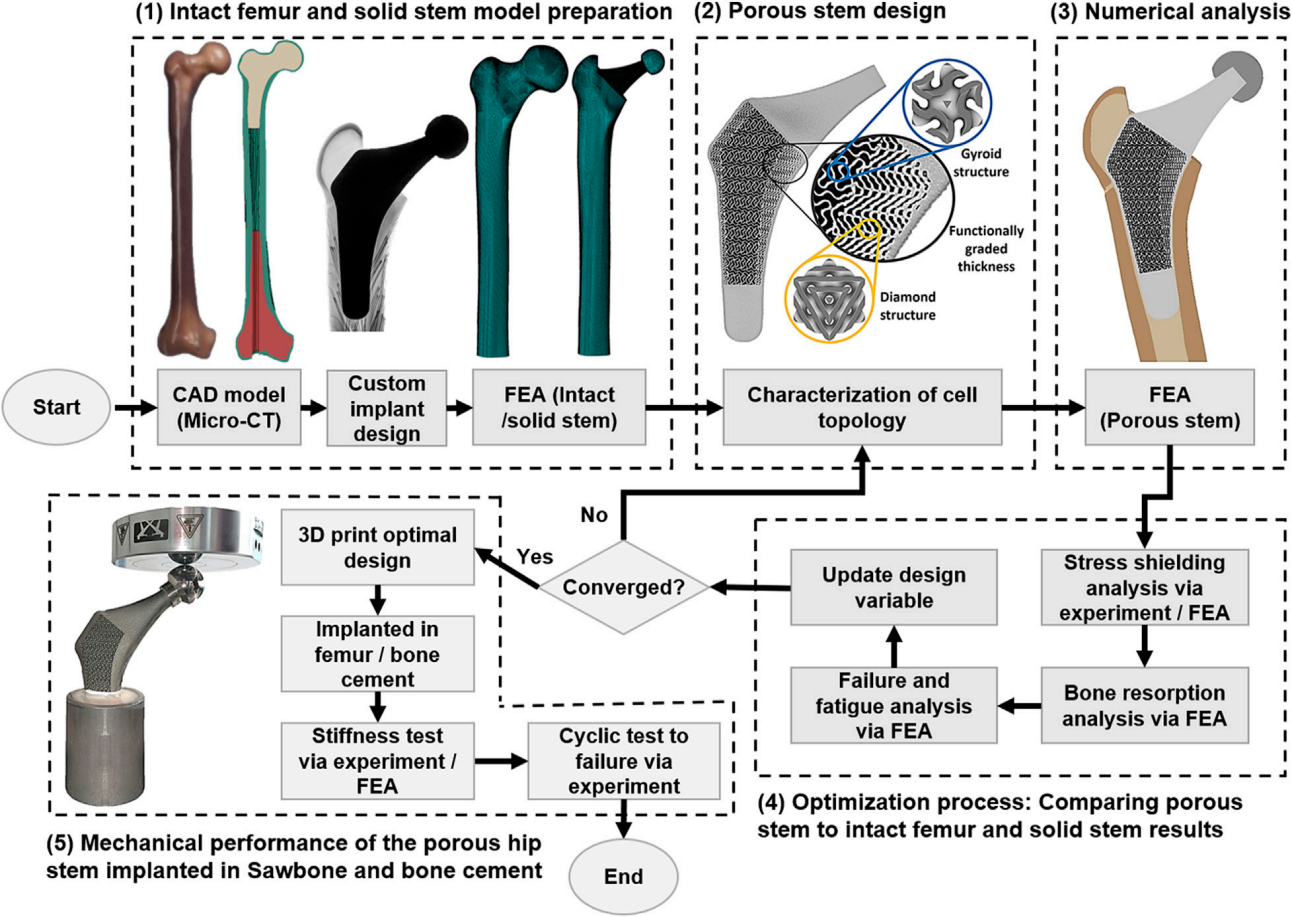
Flow chart of porous hip stem optimisation methodology.

**FIGURE 2:**
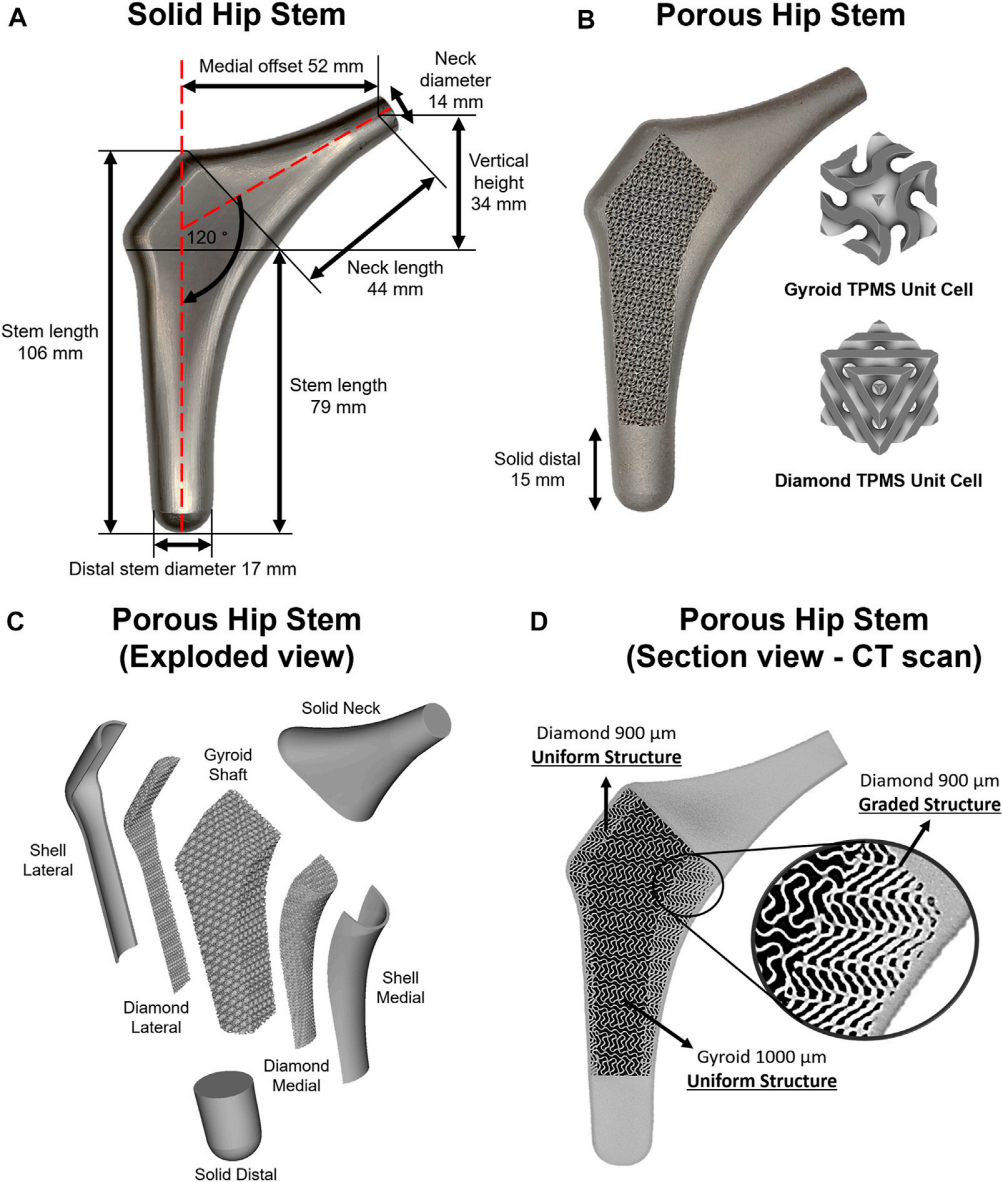
**(A)** Solid hip stem design and dimensions. **(B)** 3D printed porous hip stem with gyroid and diamond TPMS unit cells implemented. **(C)** Isometric exploded view of the porous hip stem. **(D)** Micro CT sectional view of the porous hip stem with functionally graded diamond structures within medial section.

Figure 1 illustrates the workflow of the methodology used to develop a functionally graded customised porous hip stem that reduces stress shielding. The customised hip stem was developed using a computer-aided design program SolidWorks (Solid-Works Corp.™, Dassault Systemes, Concord, MA, United States). The implant was designed for a large, left, fourth-generation artificial composite femoral bone (Model 3406, Sawbones, Pacific Research Laboratories Inc.™, Vashon, United States), and developed to have as perfectly fit as possible within the adjacent cortical bone tissue. A solid stem was manufactured from titanium using a CNC machine [BS 2TA11 (Grade 5) Ti6Al4V 20 mm thick plate 150 mm long x 100 mm wide], and consequently employed as the control group throughout this study (Figure 2A). nTopology^®^ software (version 3.25.3, New York, United States) was used for the design and development of two differing TPMS unit cells (Figure 2B) used in the hip stem geometry. The equations employed to develop Schoen Gyroid and Schwarz Diamond are shown in Eq. 1 and Eq. 2 as follows (Soro et al., 2020):

Schoen gyroid:

$$\emptyset_{G}\left(x,y,z\right)=\sin\left(\frac{2\pi }{a}x\right)\cos\left(\frac{2\pi }{b}y\right)+\sin\left(\frac{2\pi }{b}y\right)\cos\left(\frac{2\pi }{c}z\right)+\sin\left(\frac{2\pi }{c}z\right)\cos\left(\frac{2\pi }{a}x\right)=C$$ (1)

Schwarz diamond:

$$\emptyset_{D}\left(x,y,z\right)=\sin\left(\frac{2\pi }{a}x\right)\sin\left(\frac{2\pi }{b}y\right)\sin\left(\frac{2\pi }{c}z\right)+\sin\left(\frac{2\pi }{a}x\right)\cos\left(\frac{2\pi }{b}y\right)\cos\left(\frac{2\pi }{c}z\right)+\cos\left(\frac{2\pi }{a}x\right)\sin\left(\frac{2\pi }{b}y\right)\cos\left(\frac{2\pi }{c}z\right)+\cos\left(\frac{2\pi }{a}x\right)\cos\left(\frac{2\pi }{b}y\right)\sin\left(\frac{2\pi }{c}z\right)=C$$ (2)

where $\left(x,y,z\right)$ are the Cartesian coordinate system and a , b , c are the length of the unit cell in x , y and z directions respectively. Parameters a , b , and c were kept constant to obtain isotropic properties. The constant C was the defined relative density. Sheet-based TPMS structures were defined as zero-isosurface, whereby the level-set function was $\emptyset \left(x,y,z\right)=0$ . To generate a thickness on the unit cell, the unit cell domain was enclosed between two isosurfaces $\emptyset \left(x,y,z\right)=d$ and $\emptyset \left(x,y,z\right)=-d$ , where d defines the sheet thickness (Zhang et al., 2022).

The porous stem was composed of three regions and seven parts (Figure 2C). The three regions consisted of the neck, hybrid mid-stem and distal. The seven parts included the solid neck, shell lateral, diamond lateral, gyroid shaft, diamond medial, shell medial and solid distal. Both lateral and medial shells had a uniform thickness of 1 mm. Initially, the porous hip stem was designed with uniform gyroid and diamond lattice structures and consequently inserted within an artificial femur model, with a 4,839 N load applied to the femoral head. FEA was performed to obtain stress distribution on the uniform porous hip stem struts. The stress field was exported as an Excel^®^ sheet which was consequently imported into nTopology^®^ software, to optimise strut thickness for the porous hip stem, according to the stress field. The original thickness of the lattice struts was 0.3 mm. Through nTopology^®^ software, the thickness of the struts was defined based on the level of von Mises stress experienced by the struts i.e. 0.3 mm in regions where the level of von Mises stress was 0–40 MPa, a gradual increase in the thickness from 0.3 to 0.5 mm for regions where the level of von Mises stress was 40–170 MPa, and 0.5 mm in regions where the level of stresses was above 170 MPa.

Diamond lateral had a constant sheet thickness of 300 μm, and pore size and porosity of 900 μm and 56.4%, respectively (D900). The gyroid shaft had a consistent sheet thickness of 300 μm, with a pore size and porosity of 1000 μm and 67.4%, respectively (G1000). Diamond medial had a functionally graded sheet thickness (300–500 μm), founded upon the stress field and optimisation process mentioned above, having an upper-limit pore size and porosity of 900 μm and 56.4%, respectively (Figure 2D). The selected design parameters including the pore size and porosity were selected based on our previous studies (Naghavi et al., 2022c; Naghavi et al., 2022d). Pore size was defined as the inter-connected pore size, which is the diameter of a sphere that passes through the largest pore of the porous structure. To prevent stress concentration between the junctional interface of the diamond and gyroid structures, this interface was continuously blended with a 0.7 mm radius into each other through the nTopology^®^ software.

Recently, Naghavi et al. performed a detailed study characterising the mechanical properties of G1000 and D900 titanium lattice structures (see Table 2 for a summary of their findings) (Naghavi et al., 2022c). The porosity ∅ of the lattice section of the hip stem was determined according to Eq. 3, where higher values for ∅ indicated additional space for bone ingrowth. For enhanced osseointegration, ∅ had to be at a minimum of 50% (Arabnejad et al., 2016).

$$Porosity\;\left(\emptyset \right)=1-\frac{Volume\;of\;scaffold}{Volume\;of\;solid\;structure\;}$$ (3)

**TABLE 2:** Comparison of experimentally derived mechanical properties of two different scaffolds (Naghavi et al., 2022c). Dash (-) indicates absence of data.

Test	Sample name	Young’s modulus (GPa)	Yield stress (MPa)
Compression	G1000	5.68	94
	D900	10.22	159
	Cortical Bone	6–30	125–210
Tension	G1000	2.39	99
	D900	2.71	167
	Cortical Bone	2–16	77–98
Three-point bending	G1000	3.21	147
	D900	7.06	350
	Cortical Bone	3–15	45–270
Torsion	G1000	3.48	—
	D900	4.80	—
	Cortical Bone	3.1–3.7	49–98

Ti6Al4V grade 23 ELI powder (A GE Additive Company™ (AP&C)/Darwin Health Technology Co., Ltd.™, Guangzhou, China) was employed for manufacturing the porous hip stem through an SLM platform (EOS M280, Krailling, Germany). Particle size distribution was $D_{10}=$ 21 μm; $D_{50}=$ 37 μm; $D_{90}=$ 51 µm (ASTM B822) with an apparent density of 2.38 g/cm^3^ (ASTM B417). The chemical composition of Ti6Al4V powder was also investigated (ASTM B348, Table 3), containing low levels of carbon, oxygen, iron and nitrogen.

**TABLE 3:** Chemical composition of Ti6Al4V powder used in this study.

Element	C	O	N	H	Fe	Al	V	Ti
Standard values (mass%)	≤ 0.08	≤ 0.20	≤ 0.05	≤ 0.015	≤ 0.3	≤ 5.5–6.75	≤ 3.5–4.5	Balance
Measured values (mass%)	0.01	0.09	0.02	0.0022	0.22	6.44	4	Balance

Printing parameters were optimised through Darwin Health Technology Co.™ to obtain the highest print quality when manufacturing the physical porous stem from initial blueprint designs with minimal incorporated artefacts. The hatching technique used to print the hip stem was by layer stacking and fusing the Ti6Al4V powder counter-clockwise at an angle of 30° with respect to the initial position of the laser pointer. The first layer was printed at 30°, the second later was printed at 60° and the third layer was printed at 90°. This method was continued until the hip stem was manufactured completely. The details of the laser parameters are outlined in Table 4. Compressed air was blown through the lattice structure to remove any unmelted powder. The manufactured porous hip stem was removed from the build plate through a wire-cutting platform. In order to enhance lattice structure mechanical properties, samples were heat-treated at a rate of 9°C/min until a maximum temperature of 820°C, and then kept constant for 120 min, followed by acclimatisation back to room temperature within a furnace. Sandblasting was employed to smoothen the surfaces of the lattice structure with quartz sand (particle size = 50 µm/pressure = 0.6 MPa).

**TABLE 4:** Laser parameters employed for manufacturing Ti6Al4V porous hip stem.

Parameter	Laser power (W)	Layer thickness (µm)	Scan speed	Spot size (µm)	Energy density	Hatch distance (µm)
Value	190	30	1000 mm/s	90	85 J/mm^3^	110

The surface roughness of the hip stem was measured using a 4K digital microscope (VHX-7000, Keyence, Osaka, Japan) with 500 magnification, showing an average and standard deviation of 1.21 (0.62) μm, respectively. The obtained average surface roughness was within the suggested limit (1–2 μm) to enhance long term osseointegration (Wennerberg and Albrektsson, 2009).

### 2.2 Experimental protocols

#### 2.2.1 Experimental design

**FIGURE 3:**
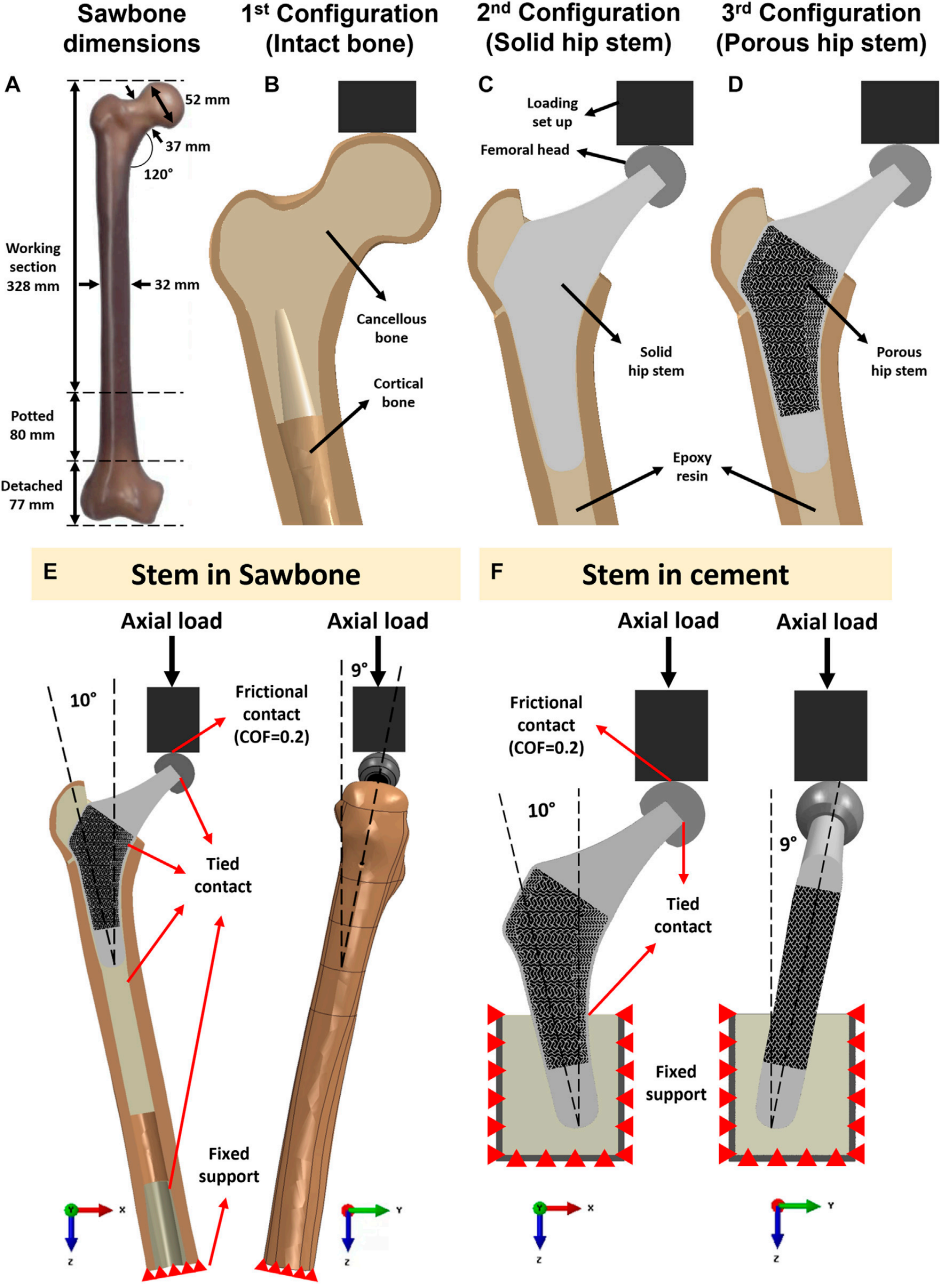
**(A)** Dimensions of the artificial femur (Sawbone). Section view schematic of **(B)** intact femur model as 1st configuration, **(C)** implanted solid hip stem femur model as 2nd configuration, **(D)** implanted optimized porous hip stem femur model as 3rd configuration. **(E)** Loading and boundary conditions of the FE model of the solid and porous hip stem fixed in Sawbone and **(F)** cement.

Figure 3 shows the three constructs that were tested in this study: an intact femur (Figure 3B), a femur implanted with a solid hip stem (Figure 3C) and a femur implanted with an optimised hybrid porous hip stem (incorporating the gyroid and diamond TPMS lattice structures - Figure 3D). A single Sawbone was used for each of the considered cases.

#### 2.2.2 Model preparation

A large, left, fourth-generation artificial composite femoral bone model (Model 3406, Sawbones, Pacific Research Laboratories Inc.™, Vashon, United States) was used in this study. The overall length of the femur was 485 mm (Figure 3A), canal diameter was 16 mm, and cortical bone had a density of 1.64 g/mL developed through e-glass fibers in combination with epoxy resin while cancellous bone had a density of 0.27 g/mL (polyurethane foam composition). Several previous studies have demonstrated that this artificial femur shows comparable biomechanical properties to human femurs (Cristofolini et al., 1996; Papini et al., 2007; Heiner, 2008).

To obtain the CAD model for the actual artificial femur bone utilised in the experiments (Figure 3A), one sawbone was scanned using a computed tomography (CT) machine (Philips™ Brilliance 64^®^ CT Scanner, Amsterdam, Netherlands). The resolution was in the range of 0.25–0.30 mm. CT images were imported into Mimics Medical Imaging Software^®^ (The Materialise Group™, Leuven, Belgium) for image processing and to develop a 3D model of the Sawbone (cancellous and cortical bones). The model was then exported into the SolidWorks CAD^®^ program (Solid-Works Corp.™, Dassault Systèmes, Massachusetts, United States).

After obtaining the CAD model of the physical femur bone, the distal condyle of the femur was dissected (by 77 mm) through a band-saw to obtain an overall length of 408 mm. The femur was potted vertically into a steel cylinder ( ∅ 100 × 80 mm) and filled with anchoring cement (Blue Circle Mastercrete Cement^®^, Tarmac Cement and Lime Ltd.™, Birmingham, United Kingdom). The final working length was 328 mm (Figure 3A). Following measurement of the surface localised strains of the intact bone (Section 2.2.3), the femoral head was resected 13 mm above the lesser trochanter at 45° (as shown in Figure 3C. Since the hip stem was designed for a perfect fit onto the cortical bone surface, nearly all cancellous bone (polyurethane foam) on the proximal medial and lateral section of the femur was removed using a surgical femur reamer. A femoral diaphyseal cement restrictor [MectaPlug^®^ (18 mm), Medacta™, Castel San Pietro, Switzerland] was also inserted prior to implanting the manufactured solid and porous stem (Section 2.1). X-Ray imaging was performed at 62 kV (DigitalDiagnost^®^ 2.1.4V22.13.567, Philips Medical Systems DMC GmbH™, Hamburg, Germany) in anterior-posterior and medial-lateral planes to verify the final position of the stem, as shown in Supplementary Figure S1. Once the hip stem position was asserted, epoxy resin (MC002568, Multicomp™, London, United Kingdom) was poured into the canal, to fill in any cavities that were preventing smooth force distribution between cortical bone and the hip stem. This allowed for enhanced force distribution from the stem onto the surrounding cortical bone, consequently enhancing FE model validation. The resin was consequently left for 24 h to solidify completely. In a clinical setting, cemented hip stems are fixed in position with bone cement with approximate density and Young’s modulus of 1.78 g/mL and 3.0 GPa respectively (Crowe et al., 2020). However, since the cure time of bone cement is very rapid and gives us less time to fix the stem in the desired position, epoxy resin with a similar density (1.69 g/mL) and Young’s modulus (2.5 GPa) as bone cement was used to fix the stem inside the femoral bone. Both bone cement and the selected epoxy resin have a cured density which falls within the established range of human bone densities, 1.18 g/mL to 1.92 g/mL making them suitable for better force distribution from the stem onto the surrounding cortical bone (Crowe et al., 2020). In all configurations (Figures 3B–D), the artificial bone was mounted according to ISO 7206–4:2010 standard.

#### 2.2.3 Strain gauge attachment

**FIGURE 4:**
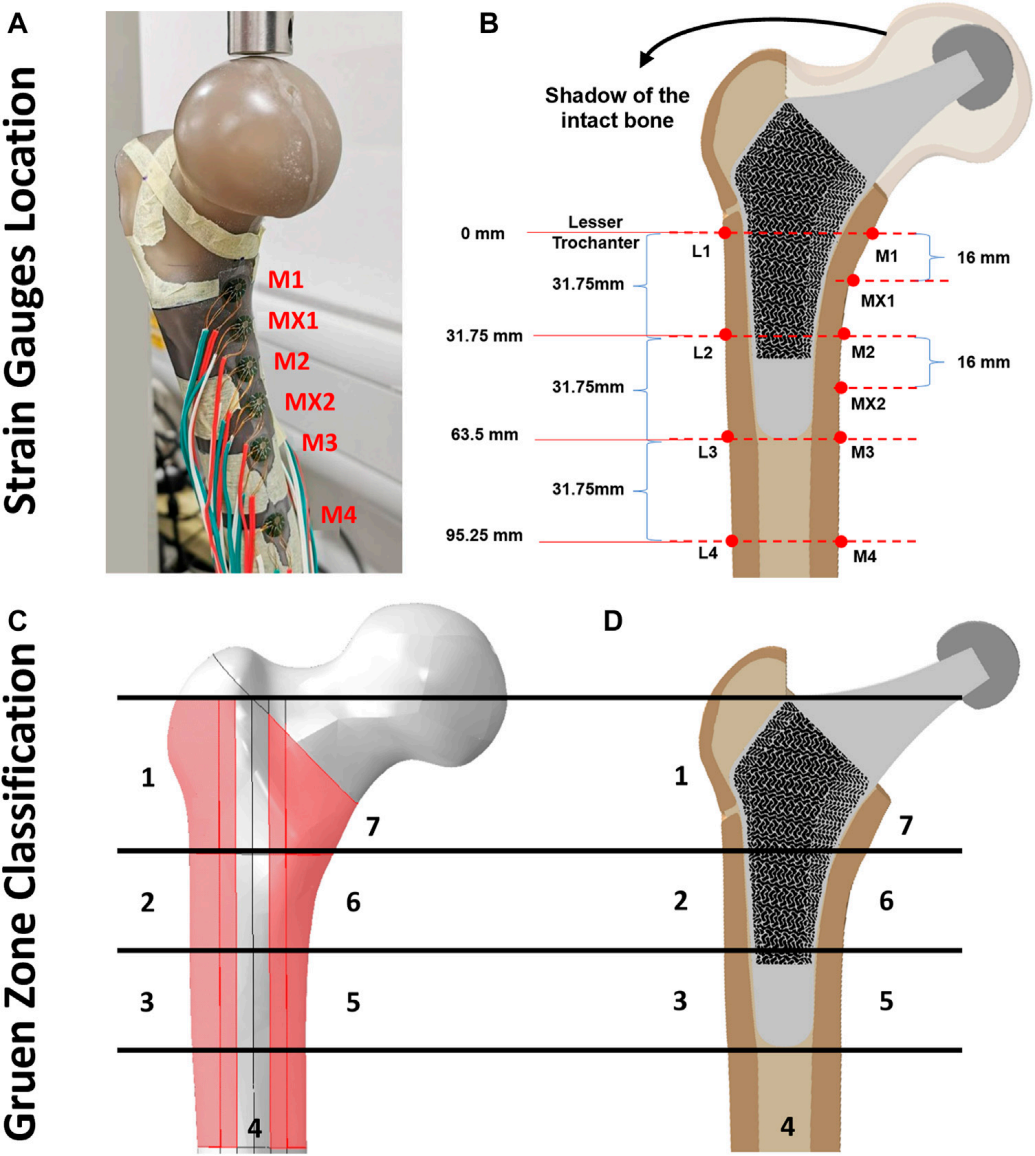
**(A, B)** Illustration of strain gauge locations across cortical bone surface, **(C)** Gruen zones (1–7) on intact femur bone model and **(D)** section view of the implanted porous stem.

The artificial femur was instrumented with 350 *Ω* rectangular rosette (45°) strain gauges (FRAB-2-350-23-1LJB-F, Tokyo Measuring Instruments Laboratory Co.™, Fukuoka, Japan) at 10 locations of interest (Figures 4A, B). Strain of the femoral bone in three different co-planar angles which results in calculating the corresponding maximum and minimum principal strains ( $\varepsilon_{\;1},\varepsilon_{\;2}$ ) and their angles w.r.t. the rosette axis; knowing the modulus of the sawbone, the corresponding stresses ( $\sigma_{\;1},\sigma_{\;2}$ ) may be calculated. Principal stresses were used to calculate the surface von Mises stress ( $\sigma_{\;von\;Mises}$ ) of the cortical bone (Eq. 4). Across all three configurations (Figures 3B–D), strain gauges were located at identical positions. Overall, six strain gauges were present on the medial region (M1-M4, MX1 and MX2), while four gauges were on the lateral region (L1-L4). Medial gauges were placed on the femur at 0 mm (M1), 16 mm (MX1), 31.75 mm (M2), 47.75 mm (MX2), 63.5 mm (M3) and 95.25 mm (M4) below the lesser trochanter, whereas lateral gauges were positioned on the femur at 0 mm (L1), 31.75 mm (L2), 63.5 mm (L3) and 95.25 mm (L4) below the lesser trochanter. All strain gauges were parallel to the long axes of the femoral shaft and were wired to half-bridge completion circuitry, preamplifier, 24-bit analogue to digital converters and serial data processor. This was connected to a laptop computer for data capturing and storage using LabVIEW^®^ software (2013, National Instruments™, Austin, Texas, United States). Mean peak count and zero-load counts were measured and subtracted for determining count variations. To obtain the local microstrain value (*ɛA*, *ɛB* and *ɛC*), the count difference was divided by the conversion value (545.4 counts/microstrain). Microstrain values were used to calculate the corresponding maximum and minimum principal strains and stresses.

#### 2.2.4 Loading and measurements

Across all three configurations (Figures 3B–D), the vertically potted femur Sawbone was distally fixed in an inclined steel platform at 10° adduction within the coronal plane and at 9° flexion within the sagittal plane (ISO 7206:2010, Figure 3E). The constructs were tested under compression, with a displacement rate of 0.01 mm/s, stepwise from 500 N up to the subclinical load of 1,200 N (corresponding to the one-legged stance phase of walking) at intervals of 100 N and holding time of 25 s each step (Ebrahimi et al., 2012). Although this load might not represent the physiological loadings for all daily activities, it assures that the construct was loaded within the linear elastic zone, preventing plastic deformation during analyses. It is shown in the literature that such artificial femurs can fail at average axial loads as low as 2,000 to 3,000 N (Ebrahimi et al., 2012). To reduce the potential effect of strength memory in the femur, a sufficient interval between the tests for each configuration was added for the relaxation of residual stress in the femur. Five technical replicate measurements were collected for excluding any potential signal errors and outliers. The mechanical assessment was performed on a Zwick machine (Zwick™ GmbH, Ulm, Germany) using a 5 kN load cell. A limitation of this study was that solely cortical bone local surface strain could be determined. However, bone resorption secondary to stress shielding was measured through the volume of identified Gruen zones (Figures 4C, D) and consequently evaluated through the finite element (FE) model.

### 2.3 FE simulation

#### 2.3.1 Component assembly

The FE model for the intact femur required validation prior to the optimisation of the porous hip implant. The femur FE model was generated based on previous CT data, with its geometry modelled within SolidWorks^®^ software (SolidWorks Corp.™, Dassault Systemes, Concord, MA, United States) (Section 2.2.2). SolidWorks^®^ was also used to generate models for the loading set-up, resin, cement potting block and the inclined platform (10° adduction in the coronal plane and 9° flexion in the sagittal plane). All models were assembled in SolidWorks^®^ and were exported in Parasolid file format (.x_t), which is easily recognized by Abaqus^®^ software (version 2019, Dassault Systèmes Simulia Corp.™, United States).

#### 2.3.2 Material properties and meshing

Material properties of the artificial femur (cortical and cancellous bone) were collected through manufacturer datasheets. Linear, elastic and homogenous isotropic material properties were assigned to the cortical shell (E = 16.7 GPa, ν = 0.3), distal and proximal cancellous bone (E = 0.155 GPa, v = 0.3), loader, femoral head and pot (E = 200 GPa, v = 0.3, stainless steel), proximal resin (E = 2.5 GPa, v = 0.3), bone cement (E = 3 GPa, v = 0.3), Ti6Al4V solid hip stem (E = 110 GPa, v = 0.3) and Ti6Al4V porous hip stem (E = 95 GPa, v = 0.3) (Ebrahimi et al., 2012).

All FE model components were meshed using a 10-node quadratic tetrahedron (C3D10; ABAQUS). A mesh convergence investigation was performed for the cortical bone model, using 25 different mesh densities. For each level of mesh density, elements of approximately uniform size were applied throughout the model, with specific element sizes ranging from 0.1 mm up to 3 mm. The solution was considered to have converged if the result did not change by > 5% for a doubling element quantity (Pegg et al., 2013). Manual mesh seeding was performed to increase the number of elements within Gruen zone regions (Figures 4C, D), where accuracy was important for experimental validation and future prediction. Concerning cortical, distal cancellous, proximal cancellous, loader, femoral head, pot, cement, solid hip stem and porous hip stem, such results converged with about 1.76, 0.41, 0.38, 0.22, 0.1, 0.1, 0.3, 9.1 and 21.5 million elements respectively. In total, the final FEA model of the intact femur (configuration 1), solid hip stem implanted femur (configuration 2) and porous hip stem implanted femur (configuration 3) had about 2.4, 11.5 and 23.9 million elements, respectively.

#### 2.3.3 Loading and boundary conditions

The interface of loading set up and femoral head was defined as a frictional contact with a coefficient of friction of 0.2 (Caouette et al., 2011). Tied contact was defined between all other surfaces, including femoral head-hip stem, hip stem-resin, resin-cortical and distal cancellous-cortical surfaces. The distal section of the femoral bone was a fixed support using an “encastered” setting, preventing displacement in all directions (Figure 3E). To validate the intact femur FE model, boundary conditions - equivalent to those employed in the experimental part of this study - were implemented (Figure 3E).

Experimental and finite element results were compared using the Bland–Altman plot. Following validation of the intact femur model additional loading scenarios were investigated, i.e., raising the load to 2,300 N and 4,839 N corresponding to activities such as jogging (Bergmann et al., 2016). Stress shielding effect and bone resorption of implanted cortical bone with the solid and porous hip stem models were modelled using the FE method under compression loading of 2,300 N.

#### 2.3.4. Stress shielding and bone resorption measurement

The measured maximum ( $\sigma_{\;1}$ ) and minimum ( $\sigma_{\;2}$ ) principal stresses from the rosette strain gauges were used to calculate the surface von Mises stress ( $\sigma_{\;von\;Mises}$ ) of the cortical bone on the predefined strain gauge locations through the following equation:

$$\sigma_{\;von\;Mises}=\left|\sqrt{{\sigma_{\;1}}^{2}-\sigma_{\;1}\sigma_{\;1}+{\sigma_{\;2}}^{2}}\right|$$ (4)

Stress shielding increase (SSI) was evaluated as the percentage difference of the von Mises stress in the cortical bone between the intact ( $\sigma_{\;Intact}$ ) and implanted ( $\sigma_{\;Implanted}$ ) femur as detailed in Eq. 5–7 below (Fraldi et al., 2010):

$$Stress\;Shielding\;Increase\;\left(SSI\right)=\frac{\sigma_{\;intact}-\sigma_{\;implanted}}{\sigma_{\;intact}}$$ (5)

$$<\sigma_{\;intact}>=\frac{1}{\sum_{e}V_{e}}\underset{V_{e}}{\sum }\underset{V_{e}}{\int }\left(\sigma_{\;e.\;intact}\right)dV$$ (6)

$$<\sigma_{implanted}>=\frac{1}{\sum_{e}V_{e}}\underset{V_{e}}{\sum }\underset{V_{e}}{\int }\left(\sigma_{e.\;implanted}\right)dV$$ (7)

Where $\sigma_{\;e.\;Intact}$ and $\sigma_{e.\;Implanted}$ are von Mises stress prior and post-THA at the centroid of each element in the femur, respectively, and $V_{e}$ is the volume of each element. SSI reflects the change in local stress within a region, post-implantation. A positive SSI implied that the local region experienced reduced stress than pre-surgical conditions, driving stress shielding. Conversely, a negative SSI, suggested a rise in local stress or potential stress concentration (Boyle and Kim, 2011).

Bone loss secondary to stress shielding was evaluated using Huiskes’ bone adaptation framework, the bone remodelling rate was described by the following formula (Pettersen et al., 2009):

$$\frac{d\rho }{dt}=\left\{\;\begin{array}{ccc} >0, & when\;S>\left(1+s\right)∙S_{intact}\; & Bone\;growth\\ =0, & when\;\left(1-s\right)\cdot S_{intact}\leq S\leq \left(1+s\right)\cdot S_{intact} & Dead\;zone\\ <0, & when\;S<\;\left(1-s\right)∙S_{intact} & Bone\;loss \end{array}\right.$$ (8)

Where $\frac{d\rho }{dt}$ is the bone density change rate, S is the strain energy density (SED) per unit bone mass ( ρ ) and $S_{intact}$ is the value of S prior to implantation (intact bone). It is known that not all the changes (overloading/underloading) in the local strain energy led to bone remodelling, and there is no bone remodelling within a certain threshold. This range is defined as the dead zone ( s ), that has a typical width value of 0.6 as obtained by Turner et al. following 2 years of clinical densitometry measurements through dual-energy X-ray absorptiometry (DEXA) (Turner et al., 2005). In this investigation, only the $S<\;\left(1-s\right)∙S_{intact}$ condition was studied, which reflects the beginning of bone remodelling and loss of bone density. General remodelling rate as a function of the strain energy density is shown in Supplementary Figure S2.

By determining the principle compressive strain prior to ( $\varepsilon_{\;intact}$ ) and post- ( $\varepsilon_{\;implanted}$ ) implantation, the strain energy ratio was calculated as:

$$\frac{S_{\;implanted}}{S_{\;intact}}={\left(\frac{\varepsilon_{\;implanted}}{\varepsilon_{\;intact}}\right)}^{2}$$ (9)

where $S_{\;implanted}$ and $S_{\;intact}$ are the strain energy post-implantation and prior to implantation respectively. Resorbed bone mass fraction $m_{r}$ of local point p could be determined through:

$$m_{r}\left(p\right)=\frac{1}{M}\int_{V}f\left(S\left(p\right)-\left(1-s\right).S_{\;intact}\left(p\right)\right)\rho \left(p\right)dV$$ (10)

where M , ρ and V are the mass, density, and volume of the intact bone, respectively. Considering $f\left(S\left(p\right)-\left(1-s\right).S_{\;intact}\left(p\right)\right)$ as $f\left(x\right)$ , we can show that:

$$f\left(x\right)=\left\{\begin{array}{c} 1,\;\\ 0, \end{array}\right.\begin{array}{c} \;when\;f\left(x\right)<0\;,\\ \;when\;f\left(x\right)\geq 0\;, \end{array}\begin{array}{c} \;Bone\;loss\\ Dead\;zone,\;Bone\;growth \end{array}$$ (11)

$f\left(x\right)$ is a resorptive function equal to unity when x<0 , while equal to 0 when x≥0 . In the case of x<0 stress shielding is present at point p and is large enough (outside the dead zone) to induce local bone resorption, whereas x≥0 suggests no bone remodelling taking place at this point. All measurements of von Mises stress and principle compressive strains were obtained and compared from each pre-defined Gruen zones (Figures 4C, D), typically employed clinically to assess THA performance.

### 2.4 Mechanical assessment of hip stem

#### 2.4.1 Static and dynamic testing

The axial stiffness for solid and porous hip stems was estimated through experimental and FE studies, followed by comparative analyses. ISO 7206–4:2010 was implemented to fix the stems using PMMA bone cement (Simplex P^®^, Stryker Corp.™, Mahwah, United States) within a cylindrical steel pot ∅ 50 × 58 mm (Figure 3F). In order to prevent bone cement cracking, an epoxy resin (MC002568, Multicomp™, London, United Kingdom) was applied above the bone cement, to cover all interfaces between the cement and the container. X-Ray image of the orientation of the potted solid and porous hip stem in the bone cement are shown in the Supplementary Figure S3.

For the physical experimental study, solid and porous hip stems were loaded up to 1,000 N load at a displacement rate of 0.01 mm/s using a Zwick machine (Zwick GmbH, Ulm, Germany), with a 5,000 N load cell. Time, load and displacement were measured with the sampling rate at 50 Hz. For the FEA investigation, a 2,300 N load was applied to the pre-defined reference point on the femoral head. In both cases, the stem stiffness was estimated based on the initial slope of the load-displacement data.

Cyclic loading was carried out on the porous stem using an Instron machine (Electroplus E3000^®^, Instron Corporation™, Massachusetts, United States) fitted with a 5,000 N load cell. The porous stem was fixed through the identical approach used for static tests. The stem was steadily loaded at a rate of 10 N/s to the mean load at 1,265 N, and then a 230–2,300 N sinusoidal cycles load (ISO 7206-4:2010) was applied with a frequency of 5 Hz. The test was set to terminate either when sample fracture occurred or upon completing 5,000,000 cycles. Time, cycle number, load and displacement were measured throughout the fatigue test with the sampling rate at 250 Hz (50 data per cycle).

It has been demonstrated that 3D-printed Ti6Al4V can last 50 million cycles at 375 MPa stress (Benedetti et al., 2016). This stress value was employed as a design criterion to optimize the porous implant within the design stage, suggesting that if the ISO standards loading conditions were considered for the porous hip stem, obtaining a von Mises stress of <375 MPa, the implant would have an infinite lifespan.

#### 2.4.2 Yield and fatigue factor of safety by FE data

To understand the stress distribution and factor of safety (FoS) for porous hip stems, different levels of physiological activities were considered in FEA, with maximum von Mises stress being determined upon porous hip stem struts. In addition, it was important to ensure local stresses upon the hip stem did not exceed the material’s yield stress ( $\sigma_{y}=$ 788 MPa) for static loading, together with endurance limit ( $\sigma_{N}=$ 375 MPa) for cyclic loading (Benedetti et al., 2016). Such loads and activities included ISO 7206–4:2010 (1,200 & 2,300 N), cycling (1,256 N), sitting down (2,935 N), standing up (3,839 N), walking (2,880 N), stance (3,340 N), climbing upstairs (3,606 N), descending downstairs (3,875 N) and jogging (4,839 N) (Bergmann et al., 2016). Yield factor of safety ( ${FoS}_{yield}$ ) and fatigue factor of safety ( ${FoS}_{fatigue}$ ) were determined as described in Eq. 12 and Eq. 13, respectively. Typically, FoS was required to be at least >2 to ensure the security of the loading system (Arabnejad Khanoki and Pasini, 2013).

$${FoS}_{yield}=\frac{Yield\;stress\;}{Maximum\;stress}$$ (12)

$${FoS}_{fatigue}=\frac{Endurance\;limit\;}{Maximum\;stress}$$ (13)

Goodman and Soderberg’s theories were employed to calculate the fatigue FoS for the porous stem (Arabnejad Khanoki and Pasini, 2013; Dharme et al., 2016). Such two theories were considered to be a conservative approach to medical applications (Liu et al., 2021a). Stress ratio (R) = 0.1, with the Minimum stress $\left(\sigma_{\min}\right)$ collected at 230 N and maximum stress $\left(\sigma_{\max}\right)$ collected at 2,300 N in one loading cycle, was applied in this case. The mean stress $\left(\sigma_{m}\right)$ and alternating stress $\left(\sigma_{a}\right)$ were determined using Eq. 14 and Eq. 15, respectively.

$$\sigma_{m}=\frac{\left(\sigma_{\max}+\sigma_{\min}\right)}{2}$$ (14)

$$\sigma_{a}=\frac{\left(\sigma_{\max}-\sigma_{\min}\right)}{2}$$ (15)

Goodman and Soderberg’s equations were employed to calculate the respective fatigue FoS using Eq. 16 and Eq. 17 **,** respectively.

$${FoS}_{\;Goodman}=\frac{1}{\frac{\sigma_{a}}{\sigma_{N}}+\frac{\sigma_{m}}{\sigma_{ut}}}$$ (16)

$${FoS}_{\;Soderberg}=\frac{1}{\frac{\sigma_{a}}{\sigma_{N}}+\frac{\sigma_{m}}{\sigma_{y}}}\;$$ (17)

where $\sigma_{ut}$ is the ultimate strength of the material (2,000 MPa). Using FE analysis, porous stem points whose ${FoS}_{\;Soderberg}$ was >2 were considered to have an infinite lifespan. However, by identifying the local points that had ${FoS}_{\;Soderberg}$ < 1 (above the Goodman or Soderberg line), we can predict and expect where the stem is deemed likely to fail due to fatigue.

## 3 Results

### 3.1 Experimental assessments and FEA model validation

**FIGURE 5:**
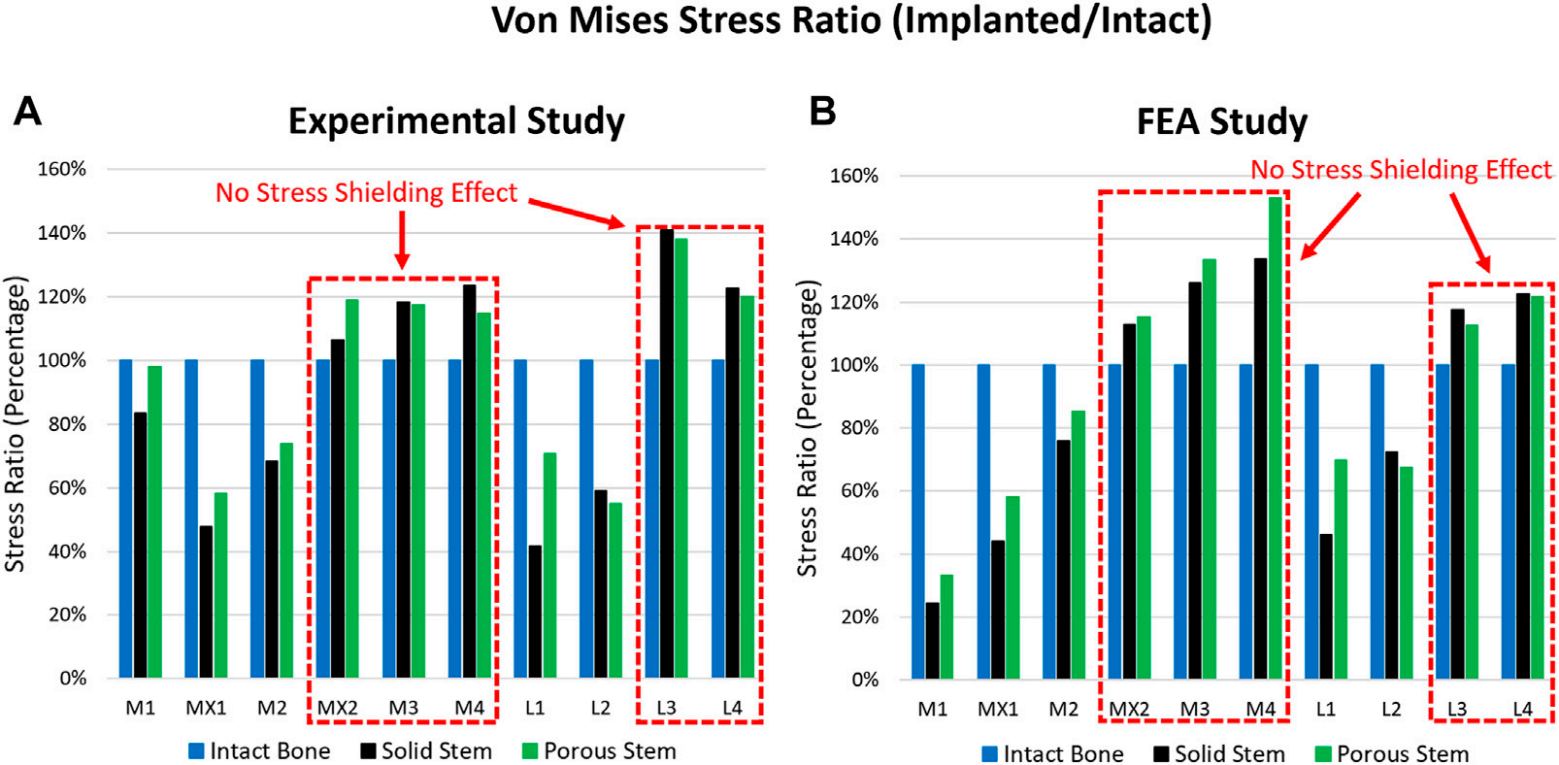
Stress ratio for 10 points of interest across the intact bone, solid stem, and porous stem, obtained through **(A)** experimental investigation, and **(B)** FE simulation.

Localised, surface von Mises stress and compressive strain forces were determined across all three configurations, through both experimental and FEA investigations. This includes data from the intact femur, femur implanted with a solid hip stem, and femur implanted with a porous hip stem. Figure 5 highlights the von Mises stress ratio for the localised strain gauge results (experimental and FEA studies). The von Mises stress ratio was defined as the von Mises stress of implanted femur divided by the von Mises value of intact bone, consequently indicating stress shifts upon each point of interest, post-implantation with solid and porous stems. Overall, a similar stress ratio trend was observed between the experimental and FEA studies, with the exception of point M1. For the medial points (M1, MX1 and M2) the stress ratio for the porous stem was higher by approximately 16% and 27% when compared to the solid stem based on the experimental and FEA data, respectively. For the lateral points, L1 had a 69% and 51% higher stress ratio based on the experimental and FEA data, respectively. L2 had a 7% lower stress ratio for both experimental and FEA data when compared to the solid stem.

When comparing the experimental and FEA stress ratio, the experimental stress ratio for the solid and porous stems in strain gauges M1 were observed to be significantly larger than the FEA stress ratio. However, the stress ratio percentage differences between the solid and porous stems (across both experimental and FEA studies) were similar, where the porous stem was higher by approximately 26%. Deviations between the experimental and FEA results were possibly due to a slight geometrical mismatch of the manufactured stems with the femur and point contact for stems in the M1 region. Limitations in manufacturing precision is known to be the cause of this geometrical mismatch that prevents full contact at the stem-bone interface. Supplementary Figure S1 shows the X-ray images of the implanted solid, porous stems inside the femur. Although both the solid and porous stems were manufactured based on the same CAD model a small gap is visible between the stem and the femur cortical shell in the distal part, in M2, L2, and L3 regions. The loose contact in M2, L2, and L3 leads to the low surface stress in these points and large stress concentration in M1, which carry the load that is supposed to be taken from the loosely contacted part of the femur. Except for point M1, experimental and FEA studies exhibited a similar pattern.

**FIGURE 6:**
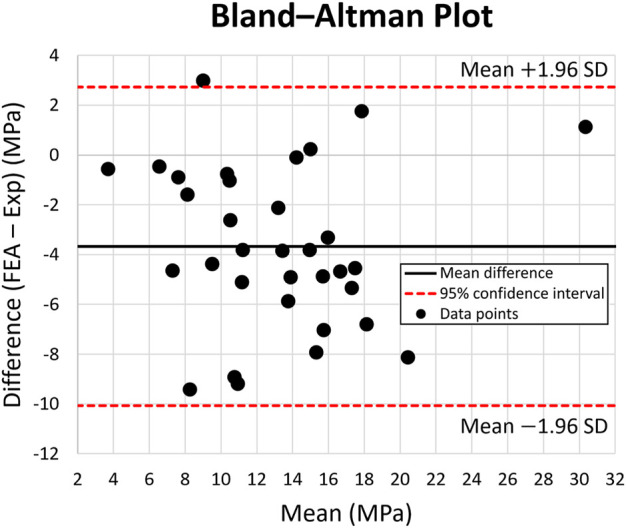
Bland-Altman Plot for von Mises stress across 10 points of interest on the intact bone, solid stem, and porous stem. The dotted lines represent 95% confidence intervals. Black data points represent the mean and variation (bias) between the von Mises stress obtained experimentally and through FE modeling, from each point of interest.

Figure 6 shows the Bland-Altman plot, used for validating the FE model vs. the experimental data. The dots present the mean and difference (bias) between the von Mises stress obtained by experiment and FEA from each point of interest. Typically, the von Mises stress obtained *via* experimental results is 4 MPa greater than the FEA results, with a 95% confidence ranging from − 10 to + 2 MPa. The Bland-Altman plot (Figure 6) demonstrated almost all data points were within the 95% confidence interval. Hence, a good agreement between the experimental and FEA results was confirmed. This enabled us to further use the FE models to test additional scenarios.

### 3.2 FEA results for simulating load at 2,300 N

**FIGURE 7:**
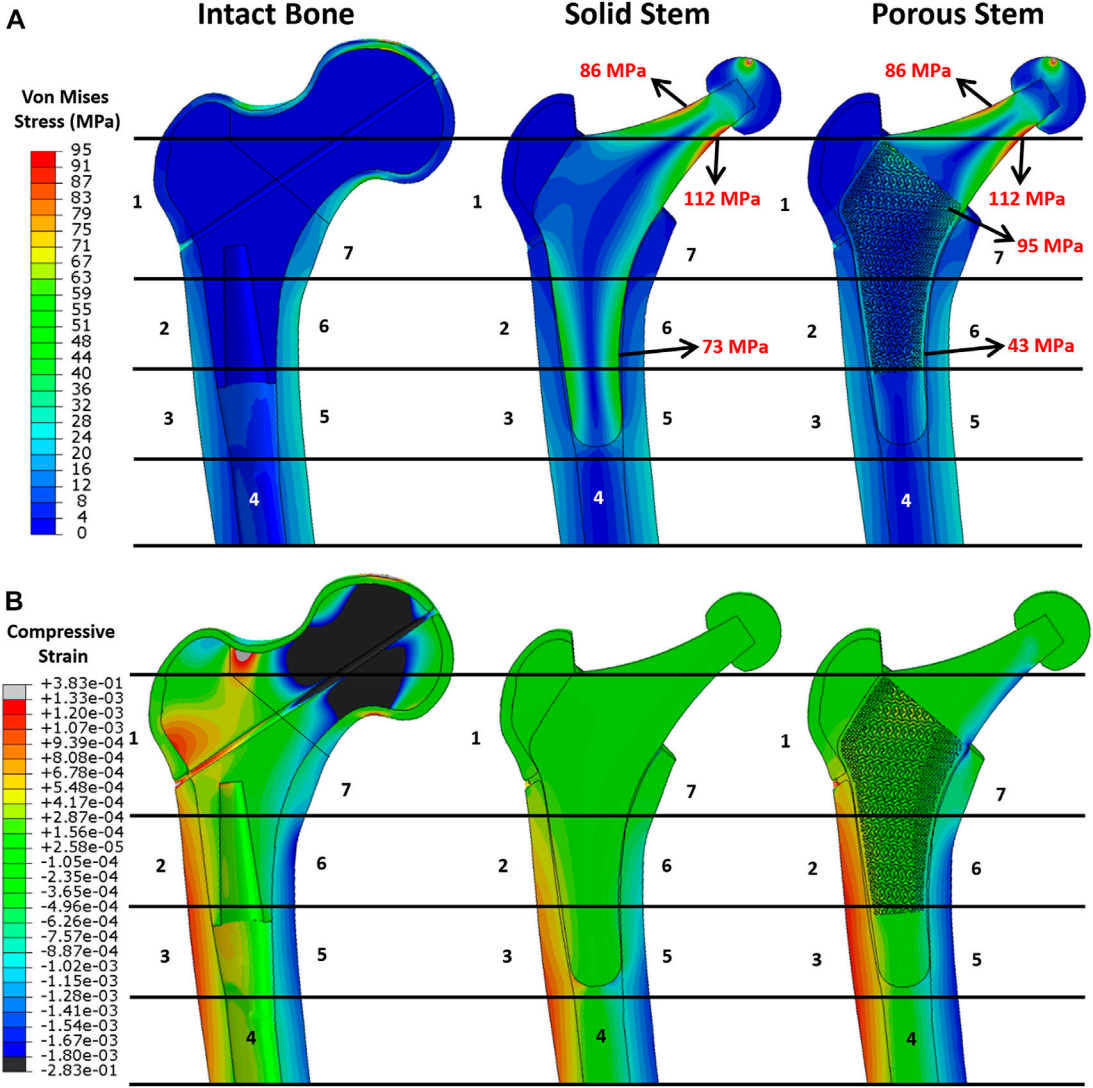
FEA results. Pattern of **(A)** von Mises stress and **(B)** compressive strain distribution across the intact bone and the implanted femur with solid and porous stems at 2,300 N load.

Once the FE model was validated, the simulating load was increased to 2,300 N, corresponding to ISO 7206–4:2010 standard. Figure 7 shows the von Mises stress and compressive strain for all three configurations. Stress distribution in the solid and porous stem neck had similar patterns, with a medial and lateral neck having a maximum von Mises stress of 112 and 86 MPa, respectively, for both stems. However, across the distal region, the level of stress in the solid stem was 73 MPa, while the porous stem exhibited a lower level of stress 43–47 MPa on the solid shell, with approximately 95 MPa being measured on the proximal struts within its porous section. The maximum von Mises stresses on the porous hip stem (112 MPa in femur and 290 MPa in cement) were effectively below the yield (788 MPa) and fatigue (375 MPa) strengths of Ti6Al4V material.

When comparing the von Mises stress on the cortical shell for all three configurations (Figure 7A), a distinctly higher stress level was observed on the femoral shaft rather than the proximal femur. This suggests that the proximal femur is the potential location for stress shielding. This stress distribution was consistent with previous research (see e.g. Hazlehurst et al., 2014). In addition, considering the Gruen zones, it was noticeable that the stress distribution in the cortical shell of the implanted femur with the porous stem were highly resembling that of the intact bone. This implies that the femur with the porous stem had a more similar pattern of stress distribution to that of the intact femur compared to that with the solid stem, that may contribute to the reduction in the stress shielding effect. Compressive strain distribution on the cortical bone is shown in Figure 7B. Positive strain indicates the extension in the local point, which is motivated by tensile stress on the lateral side of the bone. Conversely, a negative strain value indicates the compression to be motivated by compressive stress on the medial side of the bone. In the intact bone, the femur head and the lateral neck demonstrated great compressive and tensile strain respectively. Similar to stress distribution, all the configurations have large strain distributed in the distal femur, with tensile strain on the lateral side and compressive strain on the medial side. Due to the high stiffness of the solid stem, only small micro-strains were observed on the respective cortical bone in Gruen zones 1, 2, 6 and 7. It is evident that the strain values on the cortical bone with the implanted porous stem are in the identical range of strain values as intact bone. This is consistent with the findings for stress evaluation in this study.

### 3.3 Stress shielding and bone resorption evaluation

**FIGURE 8:**
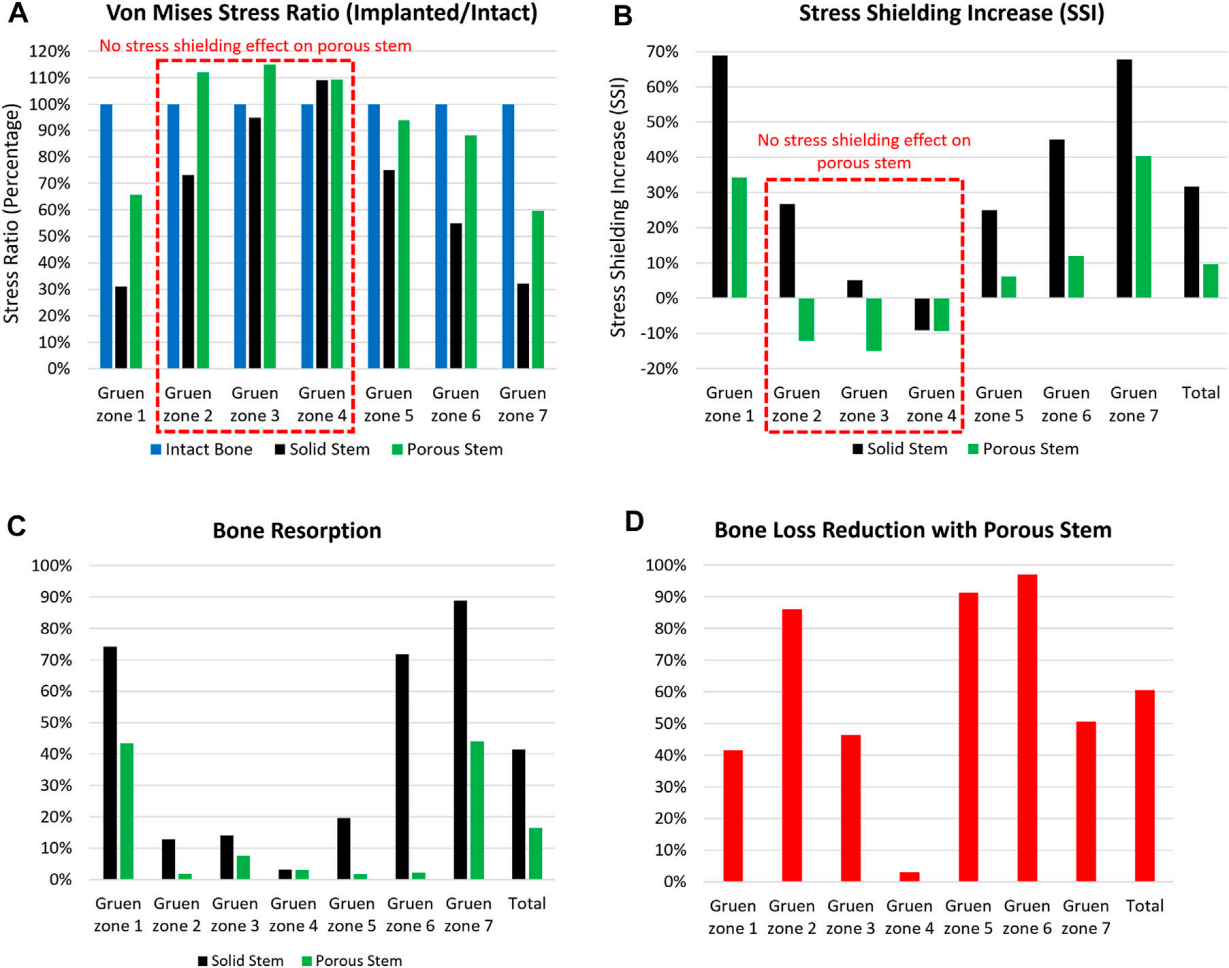
The **(A)** von Mises stress ratio, **(B)** stress shielding increase, **(C)** bone resorption, and **(D)** bone loss reduction with porous stem vs. solid stem in each Gruen zone (1–7). Total value shows the overall performance of the stem in all Gruen zone combined together.

A 2,300 N load was applied to all three configurations, with relevant von Mises stress values for each Gruen zone being recorded, averaged, and measured through FEA to calculate the stress ratio (Figure 8A). The porous stem showed more similar stress distribution to the intact bone than the solid stem, with up to 40% reduction in the von Mises stress ratio compared to the intact bone occurring in Gruen zones 1, 5, 6 and 7. Stress ratios for porous stem within Gruen zones 2, 3 and 4 were greater in comparison to the intact bone.

Figure 8B shows stress shielding increase (SSI) within each Gruen zone. A larger SSI indicates a significant reduction in equivalent stress levels within the femur post-THA, possibly leading to bone loss. For all Gruen zones (except zone 4), the absolute value of SSI for the porous stem was considerably lower in compare to the solid stem. Gruen zone 1, 6 and 7 had the largest SSI. In Gruen zone 1, the solid and porous stems had an SSI of 69% and 34%, respectively. In Gruen zone 6, the SSI for solid stem and porous stem was 45% and 12%, respectively, with porous hip stem having a reduced SSI by 74%. In Gruen zone 7, SSI was 68% and 40% for the solid stem and porous stem, respectively. This shows 41% reduced SSI for the porous stem. In Gruen zone 4, both the solid and the porous stems resulted in a similar stress value across the cortical bone, with no stress shielding effect. This finding corresponds to previous research on a porous Ti femoral stem (see e.g. Arabnejad et al., 2017). Considering the volume fraction of each Gruen zone, the total SSI value was calculated to be 32% and 10% for solid and porous stem respectively. It can be concluded that the porous stem had a 70% reduced SSI value when compared to the solid stem.

Bone loss secondary to stress shielding was quantitatively assessed by determining the resorbed bone mass fraction $m_{r}$ for both solid and porous stems within each Gruen zone (Figure 8C). For the solid stem, Gruen zone 7 was expected to have the largest bone resorption, with approximately 89% mass reduction, while Gruen zone 1 and 6 shared similar bone resorptions at ∼73%. Gruen 2, 3, and 5 had relatively less bone loss, with a mass reduction approximating 10–20%. Considering the porous stem, only Gruen 1 and 7 demonstrated significant bone resorption, at approximately 44%, while the remaining regions had a much lower bone loss, at < 7%. This trend corresponds to the stress shielding evaluation, that suggests a significant level of stress shielding might occurred in Gruen zone 1 and 7 for the porous stem (but still lesser than the solid stem). There was a limited mass reduction in Gruen zone 4 for all three stems, well in line with the previous conclusion that no stress shielding effect was observed in Gruen zone 4. Generally, the porous stem was expected to induce much less bone resorption than the solid stem in all regions except Gruen zone 4, with the total bone loss at 16% for porous stem versus 41% for the solid stem. Furthermore, according to the position of each Gruen zone, the proximal femur, including Gruen zone 1 and 7, were more vulnerable to bone loss compared to the lower part of the femur, including Gruen zone 3, 4, and 5.

To further evaluate the effectiveness of the porous stem in mitigation of bone resorption, the bone loss reduction for all pre-defined Gruen zones with porous stem were calculated and are presented in Figure 8D. The porous stem reduced the level of bone loss by 40–50% in Gruen zone 1, 3 and 7 when compared to the solid stem. Gruen zone 4 had a negligible (3%) bone loss reduction. However, remarkably, bone loss reduction with porous stem was 86–97% in Gruen zone 2, 5 and 6, suggesting that bone loss in the medial side of the femur could be successfully minimised by implementing the porous stem. Overall, the porous stem was expected to induce reduced bone resorption in comparison to the solid stem, across all regions, with total bone resorption recorded at 16%, in comparison to 41% for the solid stem. This indicates a 61% reduction in bone loss - secondary to stress shielding - for the porous stem was obtained. The amount of bone loss presented in this study is from 6 to 24 months post-operatively. Despite the fact that majority of the bone loss takes palace within the first 2 years after implantation, the reduction of bone mineral density can still continue up to 14 years after implantation. The amount of bone loss is usually detected by using dual-energy X-ray absorptiometry (DEXA) (Boden et al., 2006).

### 3.4 Stem stiffness

**FIGURE 9:**
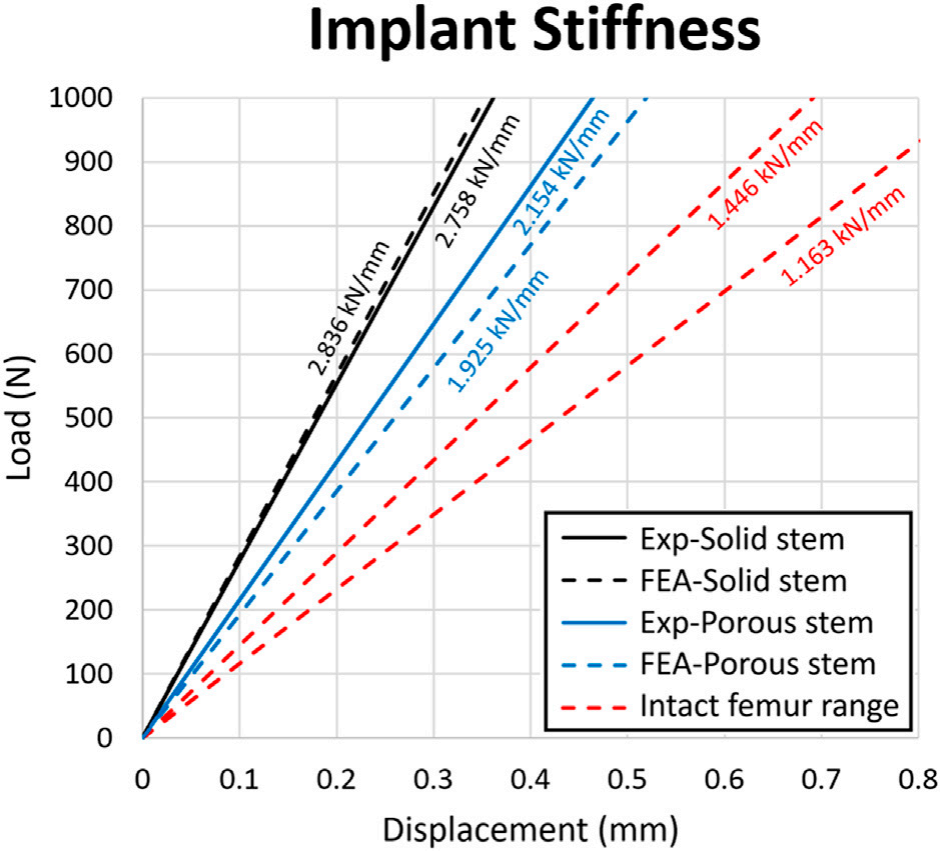
Load-displacement data for the solid stem, porous stem and intact femur. The stiffness value of each configuration was presented above the respective slopes on the diagram. Strain value corresponding to the 0.8 mm displacement was 0.01.

Figure 9 shows the load-displacement data obtained for the solid stem, porous stem, and intact femur of human (Patton et al., 2019), based on experimental and computational models. Stiffness profiles for solid stem (experimental/FEA datasets) were evaluated by linear regressions (2.76 kN/mm and 2.84 kN/mm, respectively). This represented a relative variation of 2.9%. The stiffness of the porous stem was 2.15 kN/mm (experimental dataset) and 1.93 kN/mm (FEA dataset), leading to a relative variation of 10.6%. The stiffness profile for porous stem (2.15 kN/mm) did not lie within the range of intact femur stiffness (at 1.45 kN/mm and 1.16 kN/mm for males and females, respectively), but it was closer to it than the solid stem. The relative variation between the experimental and FEA datasets could be due to differing loading/boundary conditions between the experimental and FEA investigations, including the potting level of the PMMA bone cement. PMMA Young’s Modulus (3 GPa) is significantly lower than titanium (110 GPa), which can cause additional deformation under loading. Consequently, it was expected for the experimental results to underestimate stem stiffness, which was actually observed within the solid stem dataset outcomes. Within this investigation, it was important to compare the relative stiffness of the stems rather than the results of the absolute values. Hence, major factors - including the potting material and fixation orientation of the stems - were controlled, in order to ensure the validity of all comparative analyses performed during this study. The stiffness value of the porous stem (2.15 kN/mm) was comparable (0.42–2.18 kN/mm) to the previous developed porous hip stems in the literature (Harrysson et al., 2008; Mehboob et al., 2020b; Tan and van Arkel, 2021; Al Zoubi et al., 2022).

### 3.5 Yield and fatigue factor of safety evaluation

**FIGURE 10:**
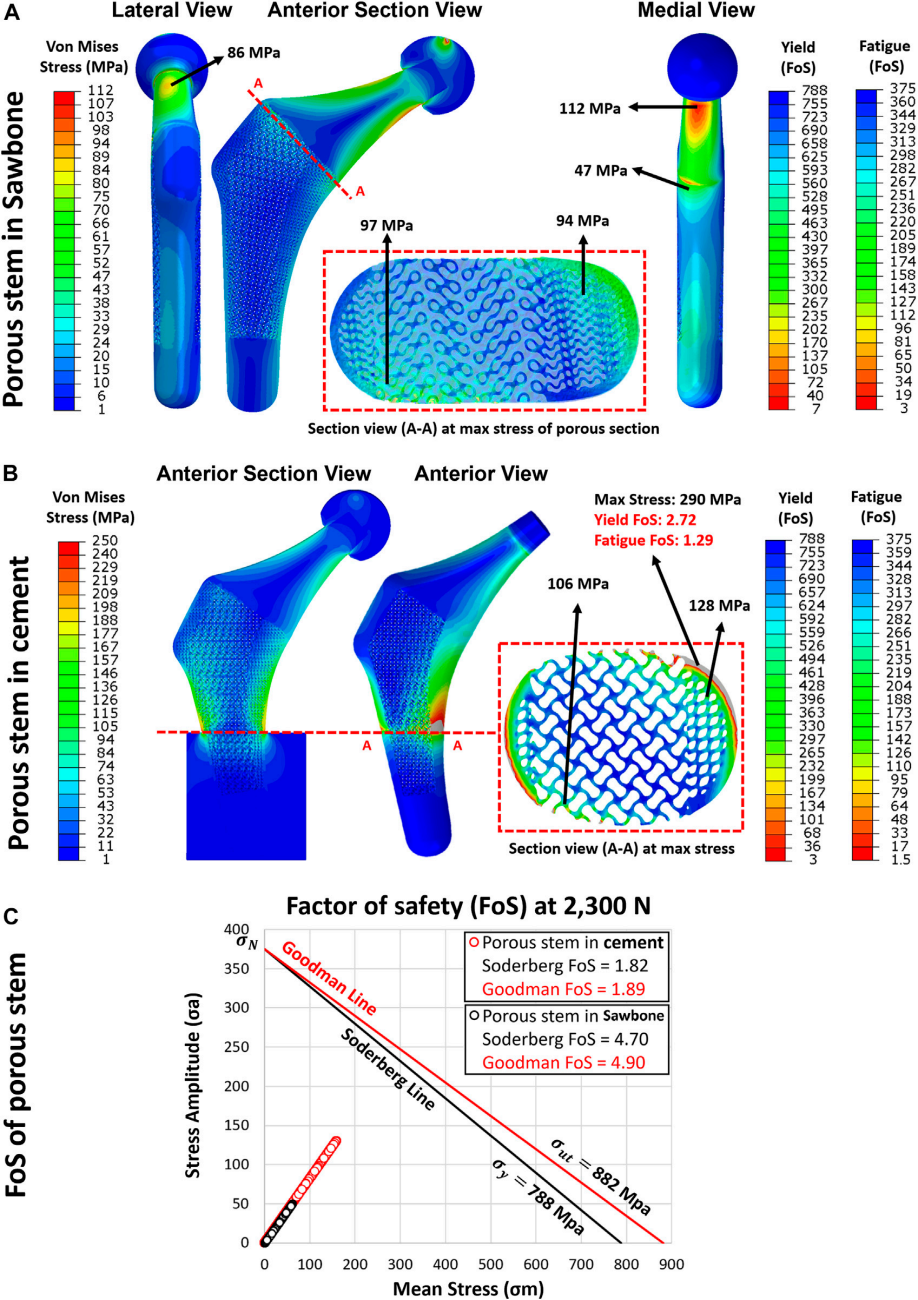
The yield and fatigue factor of safety distribution for implanted porous stem within **(A)** Sawbone and **(B)** cement, loaded at 2,300 N. **(C)** Fatigue factor of safety for each element in the porous stem implanted within Sawbone (black circle) and PMMA bone cement (red circle), examined through the Soderberg and Goodman fatigue theory loaded at 2,300 N.

Through the adoption of the FE model, the yield and FoS of each element in the porous stem were evaluated through static loading analysis. Figures 10A, B show the distribution of von Mises stress, yield FoS and fatigue FoS of the porous stem, implanted within the femoral Sawbone and PMMA bone cement, respectively. All were axially loaded at 2,300 N. When considering the porous stem embedded within Sawbone (Figure 10A), the maximum stress was observed on the medial side of the solid neck at 112 MPa. However, on the distal part (porous + shell) of the porous stem, the maximum stress was reduced to approximately 43–47 MPa on the solid shell, whereas the maximum stress on the struts of the porous section was higher, at 94 MPa and 97 MPa in the medial and the lateral sections, respectively. Within the porous section, the yield FoS and fatigue FoS were greater than 8.1 and 3.9, respectively. When considering the porous stem embedded within PMMA bone cement (Figure 10B), the maximum stress was observed upon the restricted section, on the medial shell, at 290 MPa. Within the porous section, the maximum stress was 128 MPa and 106 MPa on the medial and lateral sections respectively. Yield FoS and fatigue FoS were greater than 6.2 and 2.9 respectively for all porous stem regions.

Upon dynamic analysis, the fatigue property of the porous implant was evaluated through Goodman and Soderberg’s theories. According to the Soderberg and Goodman equation, a point in the hip stem will not undergo fatigue failure if the alternating stress and mean stress of this point both sit below the Soderberg and Goodman line. Figure 10C shows that all data points for the porous stem within the Sawbone (black circles) and the PMMA bone cement (red circle) were located well below such line and lied within the safe range. The porous stem embedded within Sawbone had a Soderberg FoS of 4.7 and a Goodman FoS of 4.9, whereas the porous stem embedded within PMMA bone cement had a lower Soderberg and Goodman FoS at 1.8 and 1.9 respectively.

Table 5 illustrates the results for maximum stress, yield FoS, fatigue FoS, Goodman FoS and Soderberg FoS for differing force levels applied to the porous stem, across varying physical activities and ISO standard levels. Only one activity (ISO-7206–4 In cement) was performed on the porous stem inside the PMMA bone cement. All other activities’ load levels were performed on the porous stem inside the Sawbone. The results suggest that in all activities, the ${FoS}_{\;Soderberg}$ ; ${FoS}_{\;Goodman}$ are all >2.2 (which is related to jogging activity at 4,839 N) except for ‘ISO-7206–4 in cement’ which had a ${FoS}_{\;Soderberg}$ and ${FoS}_{\;Goodman}$ of 1.8 and 1.9 respectively. The maximum local stresses observed on the porous stem across all physical activities (including a jogging load of 4,839 N) were all distinctly below the material’s yield stress ( $\sigma_{y}=$ 788 MPa) in static loading and endurance limit ( $\sigma_{N}=$ 375 MPa) in cyclic loading. This indicates that the implant could have an infinite service life nonetheless a higher safety factor might be required.

**TABLE 5:** Evaluation of maximum von Mises Stress, yield FoS, fatigue FoS, Goodman FoS and Soderberg FoS across varying physical activities and load levels for implanted porous stem. Activity “ISO-7206-4 In cement” was performed on the porous stem inside the bone cement with maximum von Mises stress at the distal restriction point. All other activities were performed on the porous stem inside the Sawbone with maximum von Mises stress at the medial neck of the stem (Bergmann et al., 2016).

Activity	Max. Force on hip joint	Max. von Mises stress (MPa)	Yield FoS	Fatigue FoS	Goodman FoS	Soderberg FoS
ISO-7206-4	1,200	58	13.49	6.42	9.38	9.02
Cycling	1,256	61	12.88	6.13	8.97	8.61
ISO-7206-4 In cement	2,300	290	2.72	1.29	1.89	1.82
ISO-7206-4 In Sawbone	2,300	112	7.04	3.35	4.90	4.70
Sit down	2,935	143	5.51	2.62	3.84	3.69
Stand up	3,839	187	4.22	2.01	2.93	2.82
Knee Bend	3,145	153	5.15	2.45	3.58	3.44
Walking	2,880	140	5.62	2.67	3.91	3.76
Stance	3,340	163	4.84	2.31	3.37	3.24
Stairs up	3,606	176	4.49	2.14	3.12	3.00
Stairs down	3,875	189	4.18	1.99	2.91	2.79
Jogging	4,839	236	3.34	1.59	2.33	2.24

### 3.6 Dynamic test

Supplementary Figure S4 shows the experimental setup for dynamic testing of the porous stem along with the broken hip stem. The porous stem failed where it was fixed i.e., in the cement pot after 457,349 cycles. This is while hip implants are supposed to last about 5 million cycles during their lifetime (ISO 7206-4:2010). While this can indeed be a major drawback of the porous implants, it is likely that *in vivo* application of porous implants can benefit from bone osteointegration i.e., enhancing their fatigue Nonetheless, it is possible that such implants can be more effective in the elderly population than in the younger more active population.

## 4 Discussion

Based on the experimental and FEA results, the femoral bone with a porous stem had a distinctly greater stress ratio than the femur with a solid stem, suggesting that it might be under lower risk of stress shielding. Figures 5A, B highlight that across several strain gauge locations (i.e. MX2, M3, M4, L3 and L4 - highlighted with a red dotted box), a stress ratio great than 100% (when compared to the intact bone) was obtained for both the solid and porous stems. This implies that there was no stress-shielding effect at these points. These findings are in line with the results from previous studies, suggesting limited stress shielding across the aforementioned regions (Yamako et al., 2014).

High level of stress shielding was found throughout the proximal medial and lateral section of the femur with the solid stem, reaching 69%, 45% and 68% SSI in Gruen zones 1, 6 and 7. The percentage reduction of SSI values of the porous stem compared to solid stem in Gruen zones 1, 6 and 7 were 50%, 74% and 41% respectively. In a previous study by Tan and van Arkel (2021) SSI reduction in Gruen zones 6 and 7 were reported as 15% and 25%, respectively. Corresponding values in Al Zoubi et al. (2022) were 22% and 65%, and for Cortis et al. (2022) were 11% and 25% for Gruen zones 6 and 7 respectively. Porous stem had a total SSI percentage reduction of 70% when compared to the solid stem dataset (SSI of 32% and 10% for solid and porous stem respectively). This value is significantly greater than the value of total SSI percentage reduction in previous studies ranging from 17% to 57% (He et al., 2018; Mehboob et al., 2020b; Gao et al., 2022).

Similarly, large level of bone resorption was predicted throughout the proximal medial and lateral section of the femur with the solid stem, reaching 74%, 72% and 89% in Gruen zones 1, 6 and 7. The percentage of predicted bone loss using porous stem compared to the solid stem in Gruen zones 1, 6 and 7 were 41%, 97% and 50% respectively. The total predicted bone loss for the porous stem was about 60% that is in good agreement with the value of total bone loss predicted in previous studies ranging from 40% to 75% (Arabnejad et al., 2017; Sun et al., 2018; Wang et al., 2018; Wang et al., 2020). The proposed porous hip stem showed confidence in reducing the stress shielding effect and bone resorption compared to generic solid hip stem.

Considering the stem stiffness analyses, the overall stiffness reduction of the porous stem, compared to the solid stem, was approximately 22%, based on the experimental results. The fatigue analysis of the porous hip stem showed that following the ISO standard by implanting the stems inside the femur, the porous stem can have a ${FoS}_{fatigue}$ , ${FoS}_{\;Goodman}$ and ${FoS}_{\;Soderberg}$ of 3.4, 4.9 and 4.7. The FoS mentioned for the porous stem suggests that the implant could have an infinite service life, however, following the dynamic testing, it was shown that the stem could only withstand 457 k cycles that is only around 10% of the required 5 million cycles (based on ISO-7206-4).

This study had several limitations. Numerical validation of the FE model was performed on an *in vitro* artificial Sawbone femur made of short fibers reinforced epoxy resin (simulating cortical bone) and rigid polyurethane foam (simulating cancellous bone) instead of cadaver bones. Experimental and FE models were prepared with minimal complications, causing less uncertainties from different sources. For example, a vertical load with a flat loading device was applied to the femoral head according to ISO 7206–4:2010 standard, and the effect of muscle forces and other soft tissues were not considered. Moreover, using strain gauges as a method of validating the FE model might raise concerns for validating the whole model. This is because solely local surface micro-strain could be determined on the cortical bone *via* strain gauges. Alternative way to validate the FE model was making use of digital image correlation (DIC). Manufacturing limitation of the stems resulted in a geometrical mismatch which prevents full contact of the porous stem to the surrounding cortical bone resulting in increased force concentration on the proximal medial and lateral section of the femur. Additionally, shear stress and micromotions between the stem-femur interface was not examined in this study. It is known that micromotion is more evidenced as the bone-implant stiffness mismatch is reduced (Kuiper and Huiskes, 1997). Another limitation was the use of a single artificial femur and due to cost implications, only a single solid and porous hip stem were manufactured and tested experimentally. Hence all the measurements such as stem stiffness and fatigue life of porous stem were based on that single specimen. Future studies are required to address these limitation and build on findings of this study.

## 5 Conclusion

This study investigated the stress shielding effect of Ti6Al4V additively manufactured porous hip implant versus its solid counterpart using a range of techniques. The following conclusions can be drawn:

1) A more physiological pattern of stress and strain distributions were observed with the porous hip stem when compared to the solid stem. The proximal femur (Gruen zones 1 and 7) stress shielding increase with the porous stem was only 37% compared to 68% for the solid stem, with the porous stem effectively reducing the total SSI by approximately 70% when compared to the solid stem model considering all Gruen zones 1-7.
2) In long-term use, the porous stem is expected to induce only bone resorption levels in comparison to 41% for the solid stem, meaning that the porous stem can effectively reduce bone loss within the femur by 60%. This effect was particularly distinct within the femur medial region where >90% of bone loss reduction was reported.
3) The stiffness range for natural intact femur bone (1.16–1.45 kN/mm) remained below that of both stems, though the stiffness profile of the porous stem was 22% lower than for the solid stem (experimentally determined stiffnesses were 2.15 kN/mm and 2.76 kN/mm respectively).
4) During FEA static analysis, the porous stem embedded within Sawbone was loaded up to a jogging force of 4,839 N and was observed to be safe in terms of yield FoS, fatigue FoS, ${FoS}_{\;Soderberg}$ and ${FoS}_{\;Goodman}$ , at 3.3, 1.6, 2.2 and 2.3, respectively. Corresponding values for porous stem fixed in the PMMA cement loaded at 2,300 N were 2.7, 1.3, 1.8 and 1.9, respectively. During dynamic testing, the porous stem broke from the restricted potting section of the stem following ∼457k cycles.

In summary, the current experimental setup with porous stem implanted in an artificial femur bone has shown a promising reduction in stress shielding and bone resorption. However, as part of future work, cadaveric femur with physiological loading conditions would be needed to have a better understanding of the performance of the porous stem in a more realistic scenario. For the purpose of experimentally passing the fatigue assessments - according to ISO 7206–4:2010 - further studies on the optimisation of lattice structures are required with the aim to improve the overall mechanical strength and fatigue life of porous implants. Future work can also include *in vivo* animal studies or pilot clinical studies to validate findings of this study.

## Data Availability

The original contributions presented in the study are included in the article/Supplementary Material, further inquiries can be directed to the corresponding authors.
