# Supplementary material for: A novel hybrid design and modelling of a customised graded Ti-6Al-4V porous hip implant to reduce stress-shielding: An experimental and numerical analysis
Source: Front Bioeng Biotechnol. 2023 Jan 26;11:1092361. doi: 10.3389/fbioe.2023.1092361 (PMC9910359; doi:10.3389/fbioe.2023.1092361)
Supplement: Supplementary file 1 [file DataSheet1.docx]

Supplementary Material

# Supplementary Figures


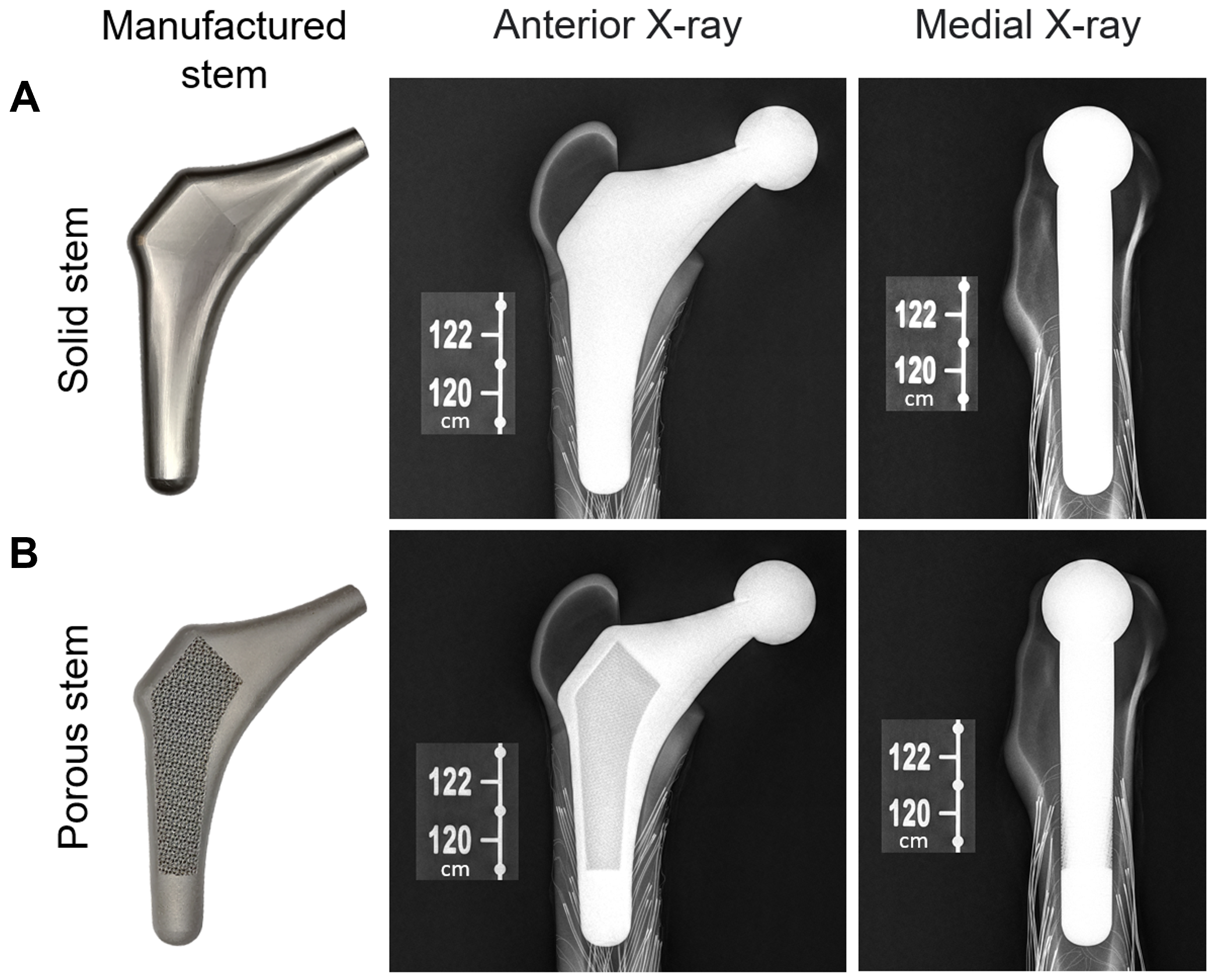


**Figure S1.** X-ray images of (A) the solid and (B) porous stems that were fixed in the Sawbone.


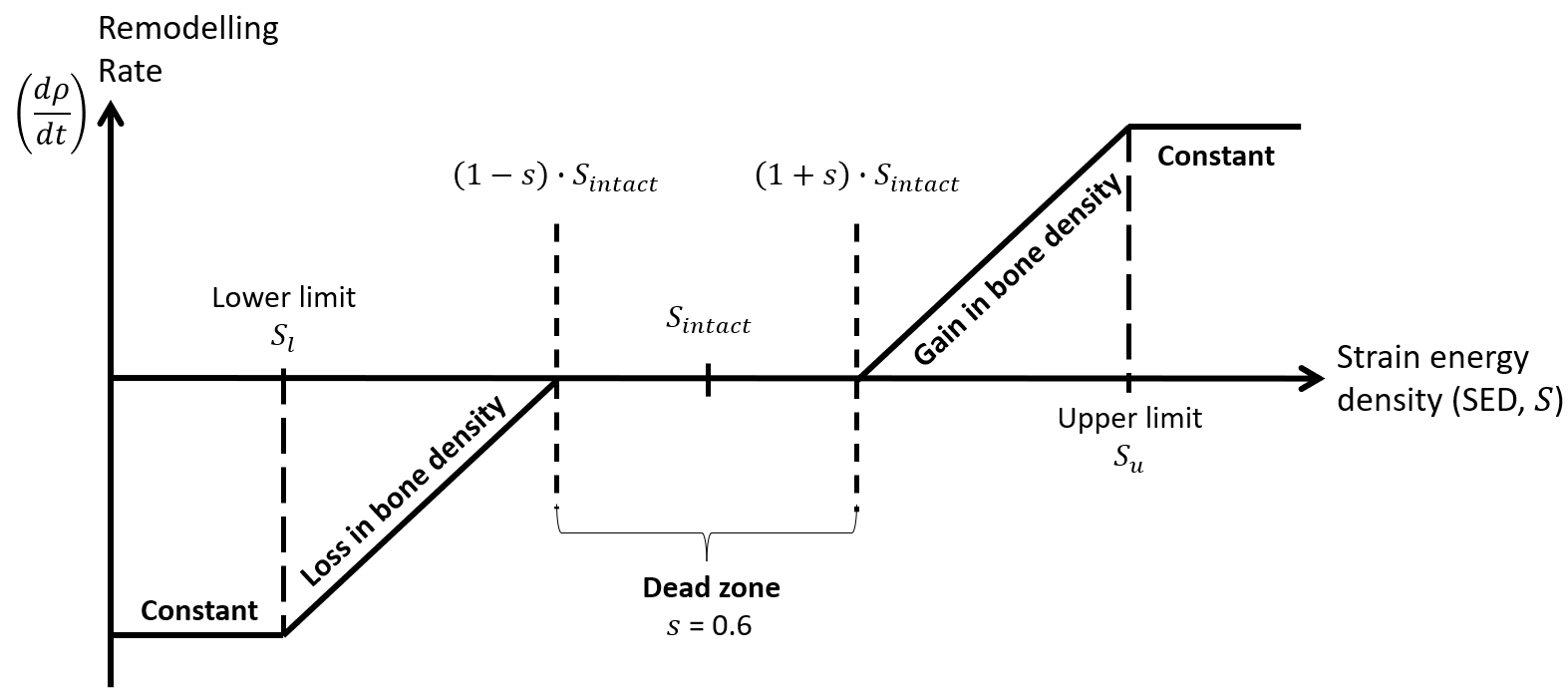


**Figure S2**. Remodelling rate as a function of the strain energy density (SED). No bone remodelling is predicted within the physiological strain range shown by the dead zone. Strain lower than dead zone causes loss in bone density, while larger strains cause gain in bone density (Turner *et al.*, 2005).


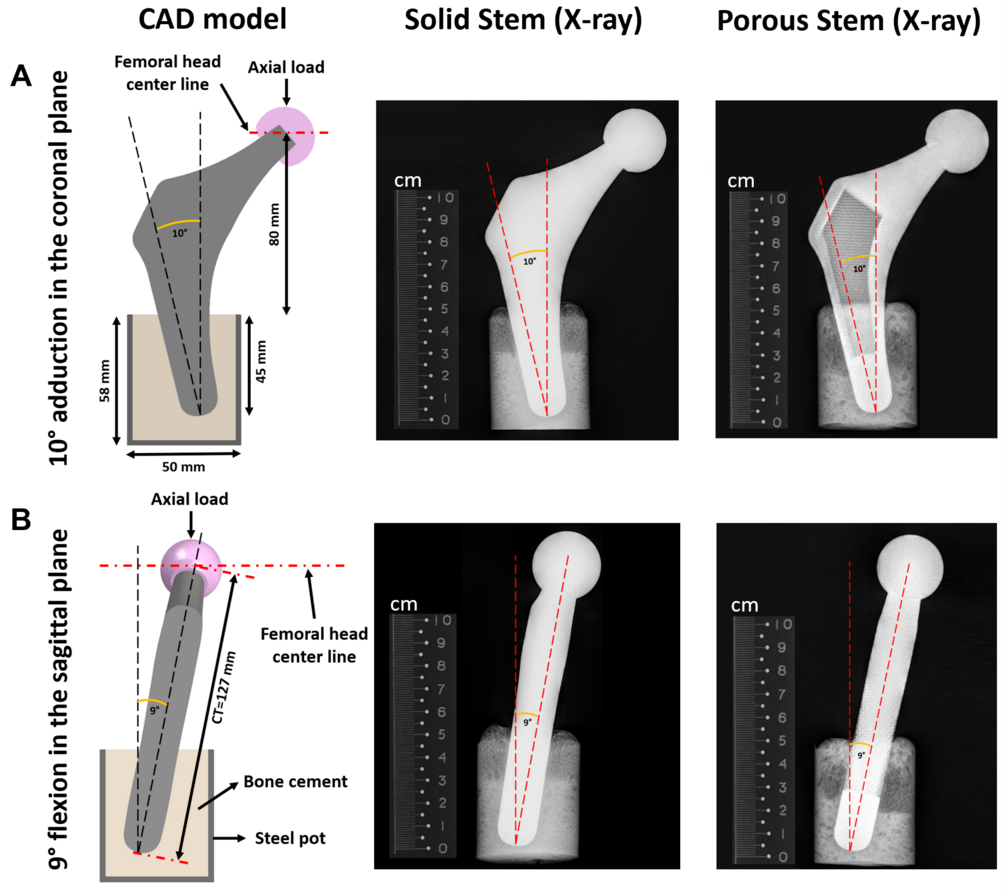


**Figure S3.** X-ray images of the (A) solid and (B) porous stems. Fixed following ISO 7206-4:2010 standard using PMMA bone cement.


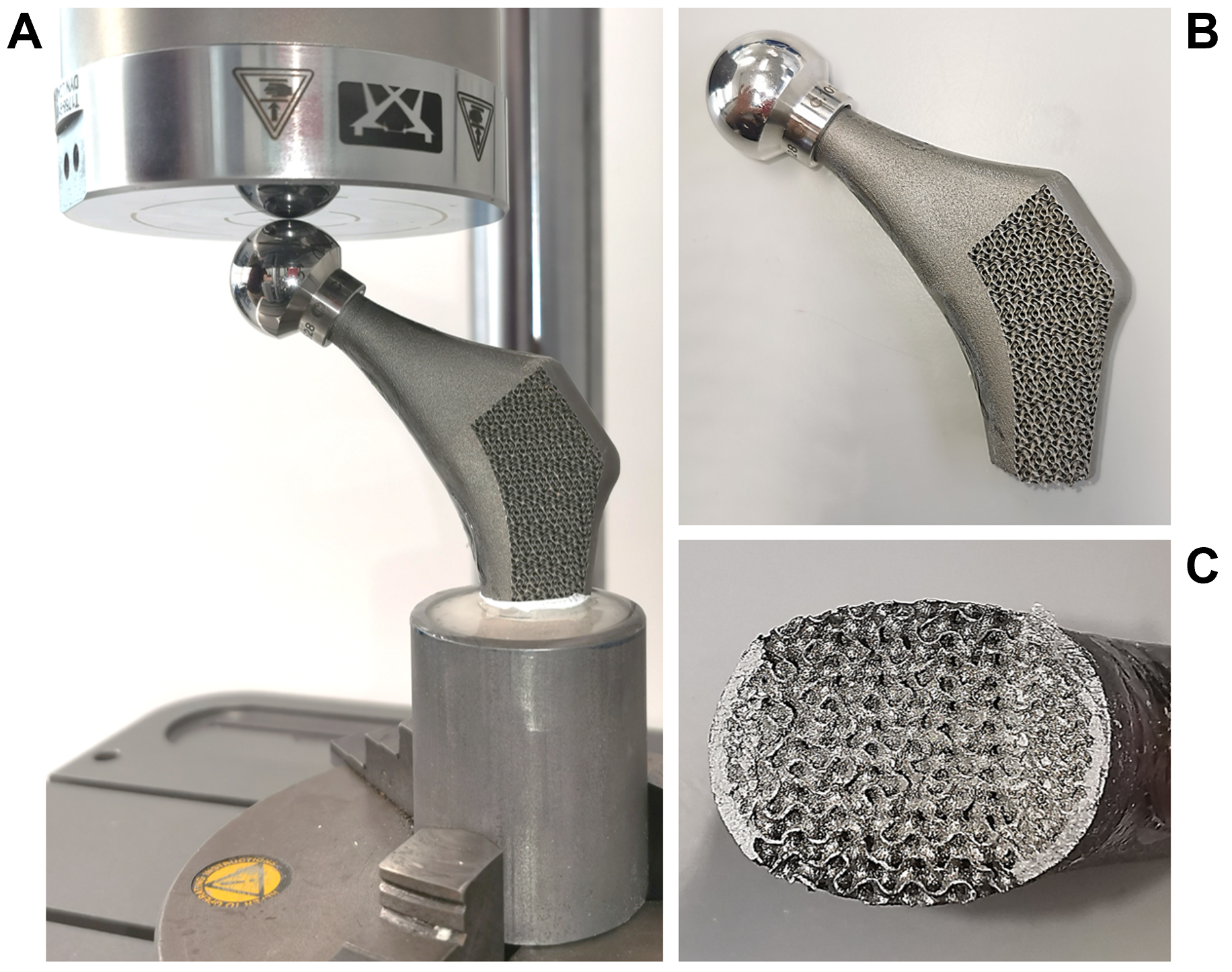


**Figure S4.** (A) Fatigue test set up used to test the porous stem. (B-C) Failed porous stem after fatigue testing.

# References

Turner, A.W.L., Gillies, R.M., Sekel, R., Morris, P., Bruce, W. and Walsh, W.R. (2005) ‘Computational bone remodelling simulations and comparisons with DEXA results’, *Journal of Orthopaedic Research*, 23(4), pp. 705–712.
